# ‘Division of labour’ in response to host oxidative burst drives a fatal *Cryptococcus gattii* outbreak

**DOI:** 10.1038/ncomms6194

**Published:** 2014-10-17

**Authors:** Kerstin Voelz, Simon A. Johnston, Leanne M. Smith, Rebecca A. Hall, Alexander Idnurm, Robin C. May

**Affiliations:** 1 Institute of Microbiology and Infection, School of Biosciences, University of Birmingham, Edgbaston, West Midlands, Birmingham B15 2TT, UK; 2 National Institute of Health Research Surgical Reconstruction and Microbiology Research Centre, Queen Elizabeth Hospital Birmingham, Birmingham B15 2TH, UK; 3 Department of Infection and Immunity, University of Sheffield, Medical School, Beech Hill Road, Sheffield S10 2RX, UK; 4 Bateson Centre, Department of Biomedical Sciences, University of Sheffield, Firth Court, Western Bank S10 2TN, UK; 5 School of Biological Sciences, University of Missouri, Kansas City, Missouri 64110, USA

## Abstract

*Cryptococcus gattii* is an emerging intracellular pathogen and the cause of the largest primary outbreak of a life-threatening fungal disease in a healthy population. Outbreak strains share a unique mitochondrial gene expression profile and an increased ability to tubularize their mitochondria within host macrophages. However, the underlying mechanism that causes this lineage of *C. gattii* to be virulent in immunocompetent individuals remains unexplained. Here we show that a subpopulation of intracellular *C. gattii* adopts a tubular mitochondrial morphology in response to host reactive oxygen species. These fungal cells then facilitate the rapid growth of neighbouring *C. gattii* cells with non-tubular mitochondria, allowing for effective establishment of the pathogen within a macrophage intracellular niche. Thus, host reactive oxygen species, an essential component of the innate immune response, act as major signalling molecules to trigger a ‘division of labour’ in the intracellular fungal population, leading to increased pathogenesis within this outbreak lineage.

## Abstract (web summary)

Outbreak strains of the pathogenic fungus *Cryptococcus gattii* display an increased ability to form tubular mitochondria. Here, Voelz *et al.* show that mitochondrial tubularization is induced by host reactive oxygen species within macrophages and facilitates rapid growth of neighbouring fungal cells.

## Graphical abstract

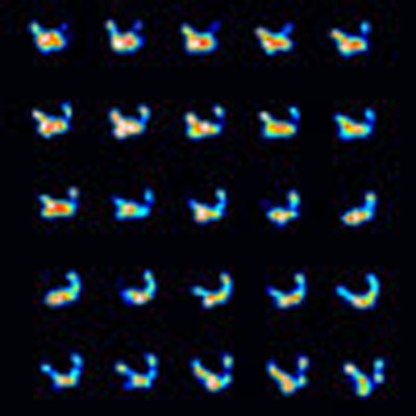

Fungal diseases are an emerging threat to human and animal health^1^,^2^. The fungal pathogens *Cryptococcus neoformans* and *C. gattii* are both causative agents of cryptococcosis, a disease with an estimated annual global burden of nearly 1 million cases and 625,000 deaths^3^. *C. neoformans* primarily infects immunocompromised patients (for example, patients with HIV/AIDS), but has also been reported in apparently immunocompetent individuals in the Far East^4^,^5^. In contrast, *C. gattii* is a primary pathogen that causes cryptococcosis in otherwise healthy individuals^6^,^7^, although rare cases in HIV/AIDS patients in Malawi and the United States have also been reported^8^,^9^.

In 1999, a *C. gattii* outbreak of fungal meningitis and meningoencephalitis started on Vancouver Island, British Columbia, Canada^10^,^11^,^12^. This outbreak has since spread to the Canadian mainland and the North-West of the United States^13^,^14^,^15^,^16^, and currently accounts for more than 400 cases^17^,^18^,^19^,^20^. *C. gattii* is divided into four separate molecular types VGI–VGIV^17^,^21^,^22^. The original outbreak on Vancouver Island was mainly caused by strains belonging to the major outbreak lineage VGIIa^12^. A second lineage VGIIb was found in the same area but shows significantly lower virulence^12^,^21^. In contrast, a later outbreak expansion to Oregon, Northwest United States, was attributed to the VGIIc outbreak lineage and exhibits similar virulence to VGIIa^14^,^22^.

There has been intense interest in explaining the origin^12^,^23^,^24^ and expansion^11^,^14^ of *C. gattii* disease, as well as characterizing features unique to the outbreak strains^25^,^26^. Our initial investigations demonstrated that outbreak strains proliferate rapidly within host macrophages, a trait that positively correlates with virulence in a mouse model of cryptococcosis. In addition, we have shown that this increased intracellular proliferation is associated with an enhanced ability of the outbreak strains to form tubular mitochondria^25^. However, the underlying mechanism causing the outbreak lineage to be virulent in immunocompetent individuals remains unexplained: a critical question from both an infectious disease and a fundamental microbiology perspective.

Here we show that reactive oxygen species (ROS), an essential component of the host innate immune response, act as a major signalling molecule to facilitate intracellular survival of this highly pathogenic fungal lineage. We demonstrate that within macrophages host ROS induce a division of labour in *C. gattii* populations, resulting in a subpopulation of intracellular fungal cells with tubular mitochondria. These quiescent *C. gattii* cells facilitate the rapid proliferation of the non-tubular pathogen population, establishing a reservoir of pathogenic fungi within the macrophage. This presents a rare example of social evolution in a pathogen, in which the costly behaviour of some individuals (entry into a non-proliferative state) confers a fitness benefit to the whole community, resulting in an overall population expansion.

## Results

### Mitochondrial tubularization is specific to outbreak strains

**Figure 1:**
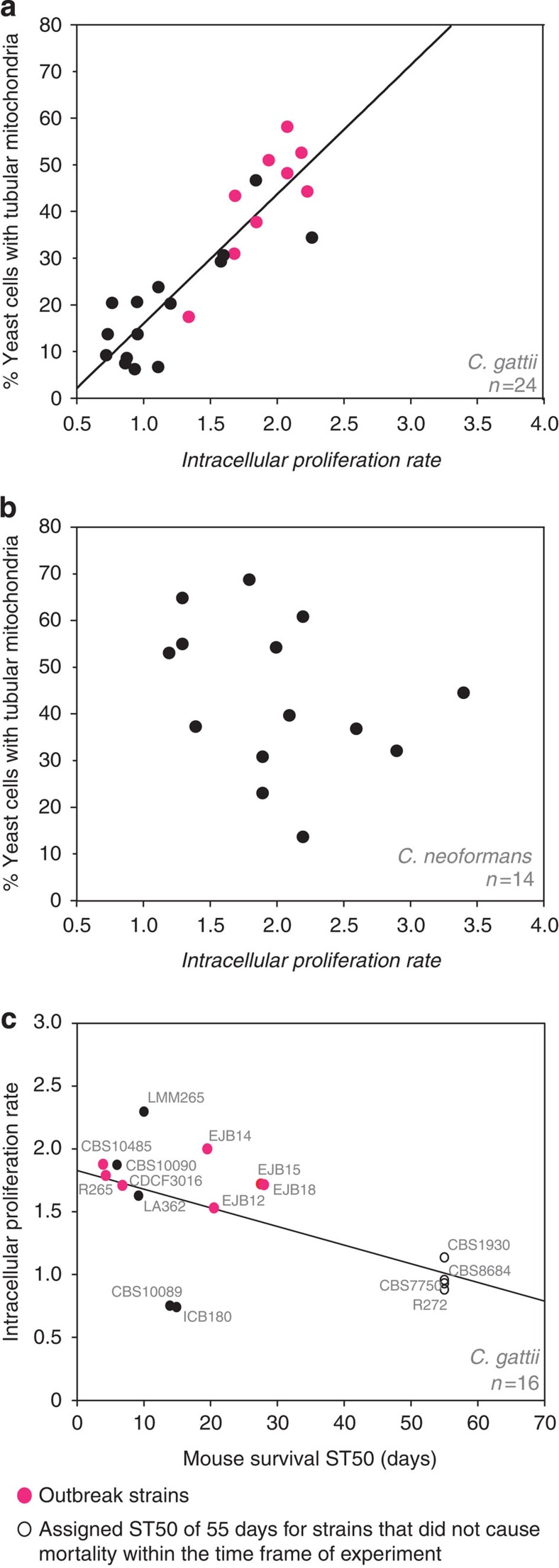
Mitochondrial tubularization is specific to outbreak strains. (**a**) The formation of tubular mitochondria in response to encounter of the intracellular niche positively correlates (linear regression) with the ability to proliferate within macrophages (Pearson correlation: *R*^2^=0.802; *P*<0.0001, *n*=24). Data were obtained for 24 *C. gattii* strains from at least four independent experiments determining IPR and three independent experiments examining mitochondrial morphology in 324–1,858 intracellular yeasts. (**b**) No correlation between yeast ability to proliferate within macrophages and formation of tubular mitochondria was observed in the opportunistic pathogen sister species *C. neoformans* (Pearson correlation: *R*^2^=0.109, *P*=0.249, *n*=14). Data were obtained for 14 *C. neoformans* strains from at least three individual experiments determining IPR and three individual experiments examining mitochondrial morphology in 1,410–3,589 intracellular yeasts (Supplementary Table 2). (**c**) Intracellular proliferation rate data significantly correlate (linear regression) with published ST50 survival data from the murine BALB/c and A/Jcr models (Pearson correlation, *R*^2^=0.356, *P*=0.015, *n*=16)^14^,^24^.

We previously reported that outbreak strains have an increased ability to proliferate within macrophages, a feature that correlates with adoption of a tubular mitochondrial morphology^25^. As this observation was based on a selected number of *C. gattii* outbreak strains, we initially tested whether this correlation is a general phenomenon of pathogenic cryptococci or whether this trait is specific to *C. gattii*. We tested 24 *C. gattii* and 14 *C. neoformans* clinical and environmental isolates from a range of molecular types (VGI, VGII, VGIII, VGIV and VNI, VNII, VNIV, respectively) (Table 1) for intracellular proliferative capacity and mitochondrial tubularization after engulfment by macrophages. The ability to switch mitochondrial morphology in concert with increased proliferation in macrophages was unique to *C. gattii* isolates (Pearson correlation: *R*^2^=0.802, *P*<0.0001, *n*=24) (Fig. 1a, Table 2 and Supplementary Fig. 1) and there was no association between tubularization and proliferation rates across 14 isolates from the related opportunistic pathogen *C. neoformans* (Pearson correlation: *R*^2^=0.109, *P*=0.249, *n*=14) (Fig. 1b and Table 2). Intracellular proliferation has previously been shown to be a good indicator of virulence^25^ and we could reproduce this correlation with intracellular proliferation rate (IPR) values and previously published ST50 survival data from both BALB/c and A/Jcr murine models (Pearson correlation: *R*^2^=0.356, *P*=0.015, *n*=16)^14^,^24^ (Fig. 1c). Taken together, mitochondrial tubularization correlates with intracellular proliferative potential and is a specific feature of *C. gattii* outbreak strains.

### Tubularization is initiated rapidly intracellularly

**Figure 2:**
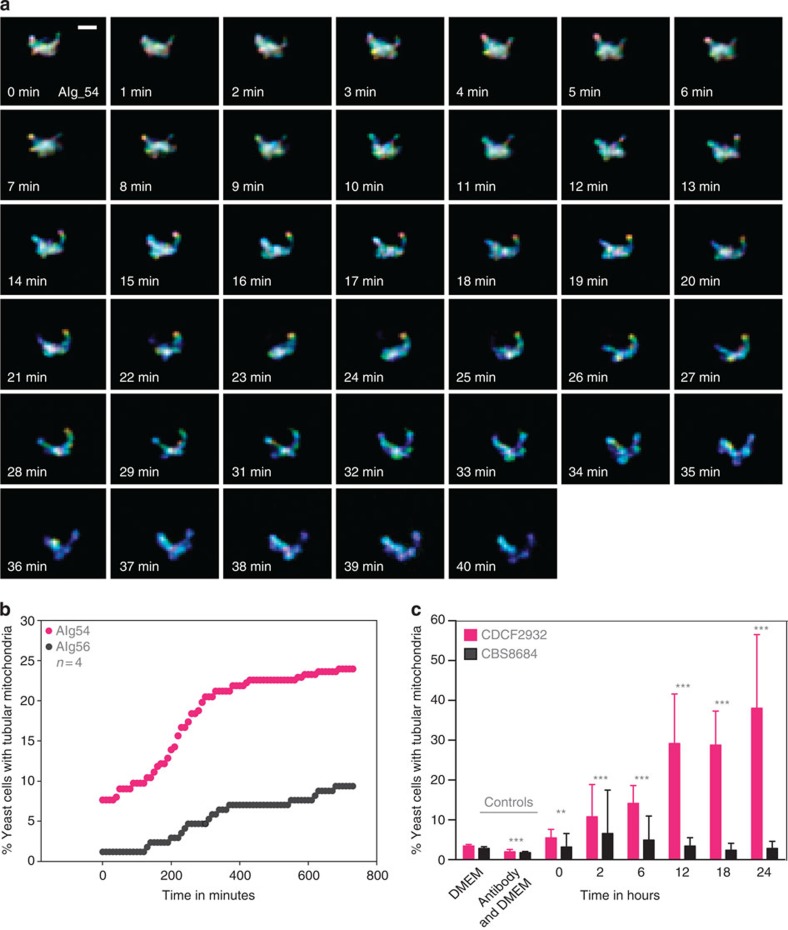
Tubularization is initiated rapidly in the intracellular niche. (**a**,**b**) The outbreak strain AIg54 (*C. gattii* R265 background) and the non-outbreak strain AIg56 (*C. gattii* CBS1930 background) were both engineered to express HEM15-GFP to visualize mitochondria. (**a**) Mitochondrial tubularization within the outbreak strain AIg54 was observed by confocal live-cell imaging. Images were generated by projecting 73 *z*-planes in a single plane with each *z*-plane given an individual colour. The globular morphology therefore gives a multi-colour unsaturated object due to the combination of many different colours and intensities over the *z*-planes, while tubular mitochondria in either the *x*- or *y*-plane give a more uniform, saturated colour. Scale bar, 1 μm. (**b**) Time-lapse analysis of AIg54 (*n*=288) and AIg56 (*n*=171) revealed fast initiation of tubularization in the outbreak strain AIg54 but a delayed and much lower tubularization response in the non-outbreak strain. Note that for both strains, absolute tubularization levels appear lower when scored by live imaging in this way than when fixed and stained with MitoTracker (Supplementary Fig. 2e), which probably reflects the higher sensitivity of the latter approach. (**c**) These results were corroborated by mitochondrial staining with MitoTracker CMXRos of the outbreak strain CDCF2932 and non-outbreak strain CBS8684 in a macrophage infection time-course experiment. Data are presented as mean average with s.e.m. Pooled categorical data (tubular versus non-tubular mitochondria) from at least three independent experiments observing between 814 and 4,114 yeasts (Supplementary Table 3) were analysed by Fisher’s exact test (**P<0.001 and ***P<0.0001). Outbreak strains are indicated by red-coloured symbols, non-outbreak strains by black-coloured symbols.

To explore the mitochondrial tubularization phenomenon further, we generated *C. gattii* strains with genetically encoded green fluorescent protein (GFP)-tagged mitochondria in either an outbreak background (AIg54) or in a closely related but non-outbreak background^25^,^26^ (AIg56), and verified that these transgenic strains were unaltered in their ability to parasitize macrophages (Supplementary Fig. 2a–d). These parental strains were chosen, as they show intrinsically similar, yet significantly different, abilities to proliferate intracellularly (outbreak strain R265 shows an IPR of 1.8±0.1, while the IPR of non-outbreak strain CBS1930 is 1.3±0.1, *n*=8, *P*=0.02 (unpaired *t*-test with Welch’s correction); mitochondrial tubularization rates are 47% for R265 and 22% for CBS1930, *n*=3, *P*<0.0001, Fisher’s exact test). Using these strains, we were able to accurately follow the kinetics of mitochondrial tubularization in individual yeast cells (Fig. 2a and Supplementary Movie 1). On encounter of the macrophage environment, yeast cells from the outbreak strain AIg54 rapidly adapted a tubular mitochondrial morphology, whereas the non-outbreak strain AIg56 was much slower to adapt and far fewer yeast cells exhibited tubular mitochondria (Fig. 2b). The same difference in adaptation was shown using an independent approach (MitoTracker CMXRos)^14^,^25^ to stain mitochondria in the AIg54 and AIg56 strains, as well as a second pair of outbreak (CDCF2932) and non-outbreak (CBS8684) strains (Fig. 2c and Supplementary Fig. 2e), confirming that tubularization was not a result of the genetic manipulation. Therefore, mitochondrial tubularization in outbreak strains is initiated rapidly after encounter of the intracellular environment.

Tubularization has previously been reported to be a protective mechanism from autophagic degradation^27^ and thus we investigated the possibility that tubular mitochondria offer protection from host autophagy. However, immunolabelling of the autophagy marker LC3 in *C. gattii-*infected macrophages revealed low numbers of LC3-positive phagosomes, averaging 2.3% (0.07–12.3%), suggesting no obvious role for autophagy in cryptococcal mitochondrial tubularization.

### Tubular yeasts are resistant and non-proliferative

Mitochondrial tubularization has been implicated in increasing the life span and fitness of cells from the fungal species *Podospora anserina* and *Saccharomyces cerevisiae*^28^, and enhancing cell viability in numerous mammalian cell types^27^,^29^. To determine any relationship between mitochondrial tubularization in *C. gattii* and intracellular fungal cell fate, we tracked individual cryptococci with GFP-tagged mitochondria within phagocytes using time-lapse microscopy. In the outbreak strain, AIg54, cells that adopted a tubular mitochondrial morphology, were killed significantly less often (Fisher’s exact test, *P*<0.0001; 288 cells, *n*=4) (Table 3). However, intracellular AIg54 yeast with tubular mitochondria proliferated significantly more slowly than those not exhibiting tubular mitochondria (Fisher’s exact test, *P*=0.0006; 288 cells, *n*=4) (Table 3). Such intracellular yeast, with tubular mitochondria and low proliferation rates, were significantly more abundant in the outbreak strain AIg54 than in the non-outbreak strain AIg56 (Fisher’s exact test, *P*<0.0001; 178 cells, *n*=4) (Table 3).

**Figure 3:**
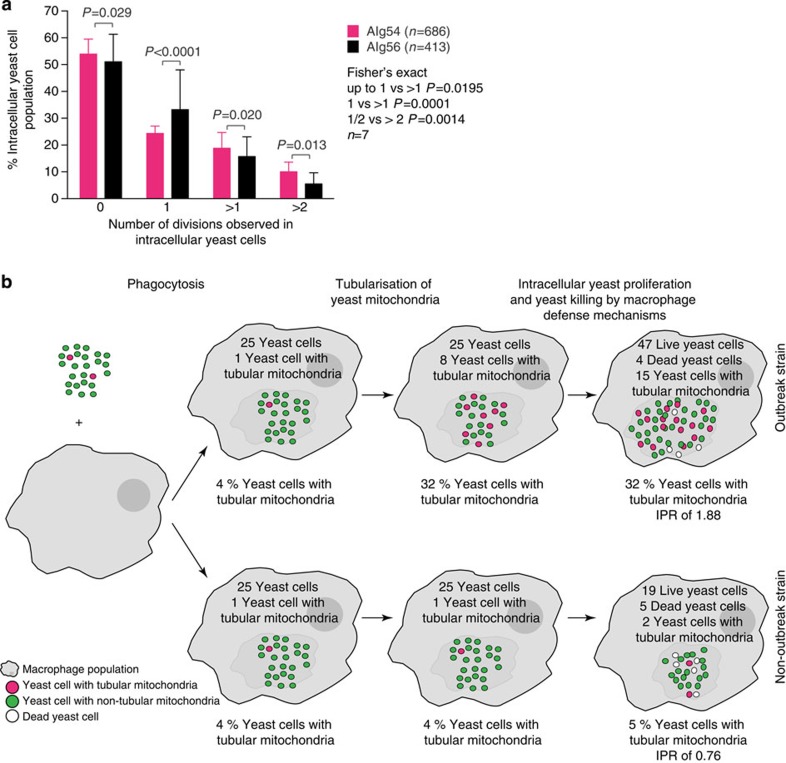
Yeast with tubular mitochondria are resistant and non-proliferative. (**a**) AIg54 and AIg56 were analysed for intracellular yeast fecundity. Intracellular AIg56 cells undergo a single division more often than intracellular AIg54 cells (Fisher’s exact test, *P*<0.0001, *n*=7), whereas intracellular AIg54 cells undergo two or more divisions more frequently (Fisher’s exact test, *P*=0.020 and *P*=0.013, *n*=7, respectively). Data were obtained from the outbreak strain AIg54 and non-outbreak strain AIg56 with HEM15-GFP-tagged mitochondria in seven independent experimental repeats examining a total of 686 and 413 yeasts, respectively, and pooled data analysed using Fisher’s exact test. Data are presented as mean average with s.e.m. (**b**) A model describing the ‘division of labour’, between a proportion of quiescent yeasts, which are essentially entirely resistant to killing by the host cell, and a small minority of ‘vulnerable’ but rapidly budding yeasts.

This observation represented a paradox: the overall number of intracellular yeast is higher at peak time post infection in the outbreak strains compared with that in the non-outbreak strains, yet outbreak strains have a higher proportion of tubular, non-proliferative cells within the population. We hypothesized that the difference may lie in the fecundity of the other (non-tubular) cryptococci within the outbreak population. Indeed, further analysis of budding behaviour of intracellular cryptococci showed that replicative non-outbreak cells typically divide only once (Fisher’s exact test, *P*<0.0001; *n*=7), whereas replicative outbreak cells (which do not adopt a tubular mitochondrial morphology) more commonly divide two or more times over the course of our time-lapse recording (Fisher’s exact test, *P*=0.0195 and *P*=0.0131, respectively; *n*=7) (Fig. 3a). Thus, within macrophages, the rapidly proliferating outbreak population of cryptococci actually represents a ‘division of labour’ between a proportion of quiescent yeasts, which are essentially resistant to killing by the host cell, and a small minority of ‘vulnerable’ but rapidly budding yeast (Fig. 3b)^30^,^31^,^32^.

### Co-infection increases non-outbreak strain proliferation

**Figure 4:**
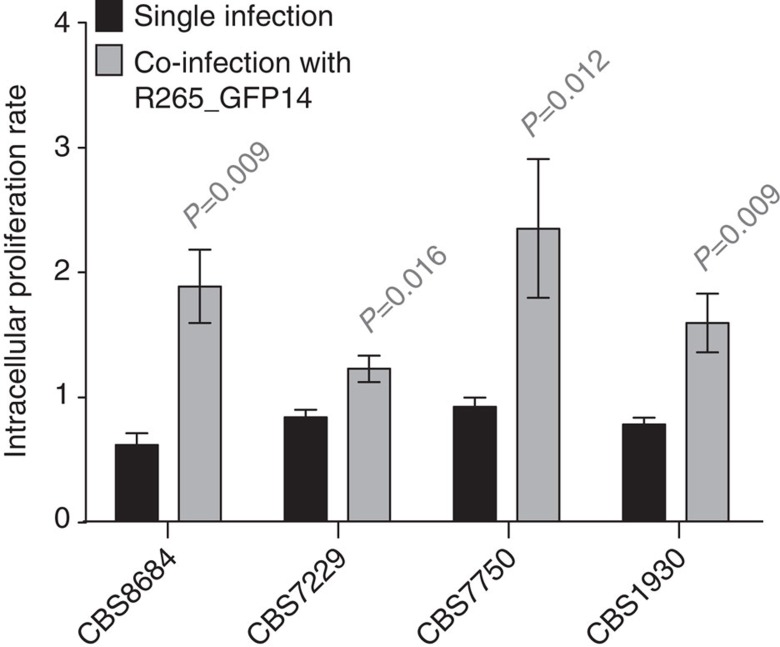
Coinfection facilitates proliferation of non-outbreak strains. The fate of intracellular yeast from non-outbreak strains inside J774 macrophages when co-infected with the outbreak strain R265_GFP14 was observed. Co-infection of non-outbreak strains with R265_GFP14 increased IPRs of non-outbreak strains. Intracellular proliferation data were obtained from five individual experimental repeats, presented as mean averages with s.e.m. and analysed by Mann–Whitney *U*-test.

As quiescent *C. gattii* cells do not themselves proliferate within the host phagocyte, we wondered whether they may confer a fitness benefit to co-infecting proliferative cells that are otherwise vulnerable to host killing. To test this hypothesis, we conducted co-infections of non-outbreak strains with the GFP-labelled outbreak strain R265_GFP14. Between 8.2% and 21.9% of phagocytes engulfed yeast cells from both strains (Table 4). Remarkably, for three non-outbreak strains tested, co-infection dramatically increased their intracellular proliferation (Fig. 4 and Supplementary Fig. 3a), while retaining the proliferative ability of the R265_GFP14 strain (Supplementary Fig. 3b,c). Thus, the presence of an outbreak strain can rescue the ability of non-outbreak strains to proliferate intracellularly. *C. gattii* virulence therefore represents a rare example of social evolution in a eukaryotic microorganism, in which the costly behaviour of a quiescent subpopulation offers a fitness benefit to the whole community^30^ and is analogous to bacterial persistence phenotypes in the context of antibiotic resistance during infection with *Staphylococcus aureus*^31^ or *Escherichia coli*^32^.

### Oxidative stress initiates mitochondrial tubularization

**Figure 5:**
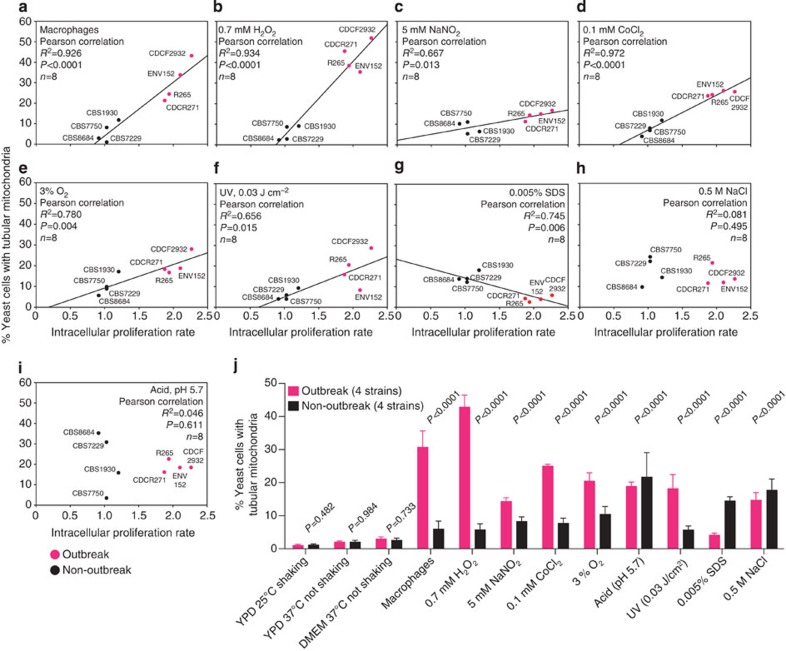
Oxidative stress initiates mitochondrial tubularization. (**a**–**j**) Yeasts from four outbreak and four non-outbreak strains, presenting with differing abilities to proliferate intracellularly, were assessed for mitochondrial tubularization on exposure to several stresses (linear regression). Treatment with 0.7 mM H_2_O_2_ (**b**) was the strongest initiator for tubularization. Data were obtained for eight *C. gattii* strains from at least nine individual experiments determining IPR and at least three individual experiments examining mitochondrial morphology in 502 to 4,859 yeasts (Supplementary Table 4). Statistical significance of correlations was assessed using Pearson correlation (**a**–**i**), whereas categorical data (tubular versus non-tubular mitochondria) between outbreak and non-outbreak strains from a set of four strains per group and three independent experiments observing between 3,402 and 23,881 yeasts (Supplementary Table 5) were analysed by Fisher’s exact test or *χ*^2^-test (**j**).

Phagocytosed pathogens are exposed to a variety of host stresses on encountering of the intracellular niche (for example, oxidative, nitrosative and hypoxic stress). Analysis of survival and growth of four representative outbreak strains and four representative non-outbreak strains under a range of stress conditions *in vitro* did not reveal any significant correlation with virulence at 2 h (Supplementary Fig. 4) or 24 h (Supplementary Fig. 5) post exposure. We therefore tested whether any of these stresses act as the stimulus to induce mitochondrial tubularization and subpopulation differentiation in outbreak strains of *C. gattii*. As previously demonstrated, there was a strong correlation between increased tubular mitochondria and increased intracellular proliferation (Fig. 5a). We hence exposed outbreak strains to individual phagocyte stresses *in vitro*, quantified mitochondrial tubularization and then tested for any association with IPR within macrophages. There was no correlation between IPR and intracellular mitochondrial tubularization imposed by environmental stresses such as low pH, cell wall stress or DNA damage (Fig. 5f–j). Several conditions such as oxidative, nitrosative and hypoxic stress that mimic the environment of phagosomes/phagolysosomes induced the formation of tubular mitochondria (Fig. 5b–e,j). Strikingly, however, exposure to a sub-lethal concentration of H_2_O_2_ (0.7 mM), which mimics the oxidative stress conditions within an early phagosome^33^, induced a mitochondrial tubularization response indistinguishable from that which occurs within phagocytic cells (Fig. 5b,j). Thus, outbreak strains of *C. gattii* probably respond to host ROS to induce mitochondrial tubularization.

The yeast p38 mitogen-activated protein kinase Hog1 is important for fungal pathogen responses to oxidative and osmotic stress within the phagosome^34^. As the regulation of Hog1 activation is dependent on the cryptococcal species^35^, we reasoned that Hog1 activation might vary between clinical and environmental *C. gattii* isolates. Therefore, Hog1 activation in response to oxidative and osmotic stress was determined for two outbreak and two non-outbreak isolates. However, the dynamics of Hog1 activation in *C. gattii* were similar to those previously published for *C. neoformans*, with no observable differences detected between outbreak and non-outbreak isolates (Supplementary Fig. 6). Therefore, differences in activated Hog1 levels are not responsible for the observed increase in *C. gattii* mitochondrial tubularization.

### Reducing host ROS decreases outbreak strain proliferation

**Figure 6:**
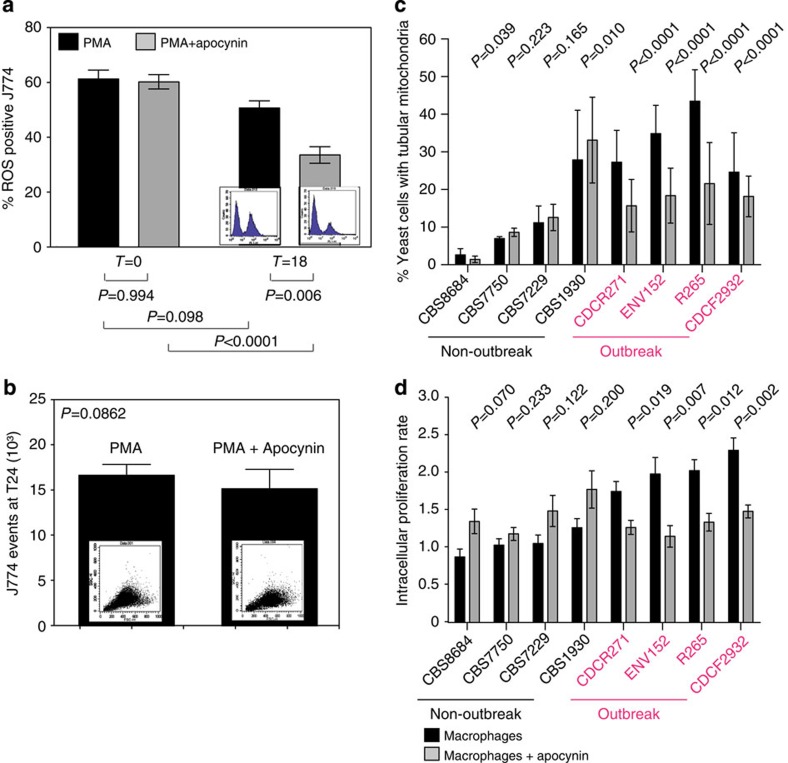
Reducing host ROS decreases proliferation of outbreak strains. (**a**,**b**) Treatment with 0.5 mM of the NADPH oxidase inhibitor Apocynin reduced the amount of ROS-positive J774 macrophages after 18 h (*P*=0.0006). (**a**) ROS production by macrophages was measured by flow cytometry after cell staining with 2′,7′-dichlorodihydrofluorescein diacetate. (**b**) Macrophage survival was not affected by the inhibitor. Data were collected from four independent experiments, presented as mean averages with s.e.m. and data analysed with Mann–Whitney *U*-test. (**c**,**d**) Inhibition of ROS reduced the proportion of intracellular yeasts presenting with tubular mitochondria in outbreak strains and significantly reduced the ability of the outbreak strain to proliferate intracellularly. Data were obtained for eight *C. gattii* strains (four outbreak and four non-outbreak) from nine individual experiments determining IPR, presented as mean averages with s.e.m. and analysed by Mann–Whitney *U*-test. For assessment of mitochondrial morphology, three individual experiments examining mitochondrial morphology in 914 to 2,209 yeasts (Supplementary Table 6) were analysed and these categorical data assessed by Fisher’s exact test.

Given the probable role of ROS in inducing mitochondrial tubularization, we hypothesized that reducing host ROS production may cause outbreak strains to be unable to respond appropriately to the intracellular niche. By using a low dose (0.5 mM) of the NADPH oxidase inhibitor apocynin, we were able to significantly reduce ROS production by macrophages without affecting phagocyte survival (Mann–Whitney *U*-test, *P*=0.006 and *P*=0.862, respectively; *n*=4) (Fig. 6a,b). Under these conditions, both mitochondrial tubularization and intracellular proliferation were significantly reduced in outbreak strains, but remained unaltered in non-outbreak strains (Fig. 6c,d and Supplementary Fig. 7a–c). To understand how apocynin was modulating the behaviour of *C. gattii* outbreak strains during macrophage infection, we followed individual intracellular fungal cell fates with time-lapse microscopy, as described above, in the presence and absence of apocynin. This demonstrated that the number of proliferating cells dropped by 20.4% in the outbreak strain AIg54 (Fisher’s exact test, *P*=0.041, *n*=3) but was not significantly changed in the non-outbreak strain AIg56 (increase of 7.5%, fisher’s exact test, *P*=0.570, *n*=3). There was a corresponding increase in quiescent cells (Fisher’s exact test, *P*=0.030, *n*=3) but no change in the proportion of the population that was killed (Fisher’s exact test, *P*=0.517, *n*=3), suggesting that the reduction in the size of the tubular subpopulation reduces the ‘division of labour’ in outbreak strains and limits the ability of the population as a whole to proliferate intracellularly. Interestingly, this effect was not seen in co-cultures grown *in vitro* in the presence of 0.7 mM H_2_0_2_ (Supplementary Fig. 7d,e), suggesting that ROS-induced mitochondrial tubularization induces a pathogen response that protects cryptococci from their macrophage host rather than directly conferring resistance to oxidative damage.

## Discussion

We here present findings that describe how a fungal outbreak lineage of *C. gattii* uses host antimicrobial mechanisms, that is, the generation of ROS, as a signal to induce an intracellular survival and proliferation programme within macrophages. This programme induces a proportion of the fungal cell population to adopt a quiescent state that is characterized by tubular mitochondria and resists killing in the intracellular niche, while the remaining population is vulnerable to killing, but proliferates more rapidly, leading to an overall population expansion that overwhelms the host.

Mitochondria are involved in a wide range of processes encompassing cellular stability, energy production, stress resistance and regulation of life span, and hence are key in determining the fitness of microorganisms. In fact, mitochondria have been associated with virulence in several pathogens. Olson and Stenlid^36^ described the control of virulence by the mitochondrial genome in the fungal plant pathogen *Heterobasidion annosum*. In addition, mutants with dysfunctional mitochondria in the human fungal pathogens *Candida glabrata* (which also proliferates intracellularly in macrophages), *C. albicans*, *C. neoformans* and *Aspergillus fumigatus* all show attenuated virulence in mouse model systems of disease^37^,^38^,^39^,^40^,^41^,^42^. However, to date, the direct molecular mechanisms regulating mitochondrial involvement in virulence are not understood.

Several studies have shown the importance of numerous mitochondrial functions in conferring virulence by activating genes involved in metabolic pathways^37^,^43^, mitochondrial respiration^44^ and survival response to oxidative stress^40^,^41^,^45^,^46^. This is suggestive of a global stress response to the harsh environment within the host, and hence it is likely to be that mitochondrial tubularization might be a marker of global stress responses rather than the protective mechanism itself. In support of this, we have recently shown that transfer of a mitochondrial ‘outbreak’ genotype is necessary, but not sufficient, to confer outbreak virulence phenotypes between lineages^26^, indicative of the complex, multigenic nature of the outbreak phenotype.

In summary, here we introduce a new model for virulence in outbreak strains of *C. gattii*, driven by a division of labour between an intracellular yeast subpopulation that adapts a quiescent and resistant tubular mitochondrial phenotype and a susceptible, but highly proliferative, non-tubular mitochondrial yeast subpopulation. The phenomenon of persister cells has been described for bacterial pathogens to explain latent bacterial infections and antibacterial resistance^47^. In addition, very recent data have shown that bistable expression of virulence genes in *Salmonella typhimurium* creates phenotypically heterogeneous virulent and avirulent subpopulations^48^. There is an ongoing debate about latency and recurrence in cryptococcosis^49^, and in this context a quiescent subpopulation might argue for cryptococcal latency and the risk of developing antifungal resistance^50^. In addition, the ability of outbreak strains to facilitate the proliferation of non-outbreak strains within phagocytes poses important questions regarding the impact of *C. gattii* infections on altering susceptibility to other bacterial, viral or fungal pathogens.

## Methods

### *Cryptococcus* strains, yeast and cell line culture

*Cryptococcus* strains (Table 1) were cultured in liquid culture containing 1% peptone, 1% yeast extract and 2% D-(+)-glucose (YPD) at 25 °C rotating at 20 r.p.m. 24 h before use in experiments^51^.

The semi-adherent macrophage-like cell line J774 (European Cell Culture Collection) was used for macrophage experiments^52^. The cells were used between passage 5 and 15 after thawing and cultured in low glucose DMEM medium (Sigma) supplemented with 10% heat-inactivated fetal bovine serum (FBS), 2 mM glutamine, 100 μg ml^−1^ streptomycin and 100 units ml^−1^ penicillin (culture media) at 37 °C and 5% CO_2_ (refs 8, 14, 51, 53, 54). Human primary peripheral blood monocytes were obtained from independent healthy volunteers. Consent was obtained from all subjects under ethical approval granted by the University of Birmingham Research Ethics Committee. Mononuclear cells were isolated over a Ficoll-Plaque PLUS (GE Healthcare) gradient. Blood samples were diluted twofold with PBS (pH 7.2) and 30 ml was centrifuged over a 20-ml Ficoll-Paque PLUS cushion at 400*g* for 30 min without braking. The mononuclear layer was collected and washed with PBS to remove platelets. Monocytes were isolated by adherence to plastic at a concentration of 4–6 × 10^6^ cells per ml in RPMI 1640 media supplemented with 2% heat-inactivated FBS, 2 mM glutamine, 100 μg ml^−1^ streptomycin, 100 units ml^−1^ penicillin at 37 °C and 5% CO_2_ for 1 h. Non-adherent lymphocytes were removed with warm PBS and adherent cells differentiated into macrophages in RPMI 1640 supplemented with 10% heat-inactivated FBS, 2 mM glutamine, 100 μg ml^−1^ streptomycin, 100 units ml^−1^ penicillin (culture media) containing 100 units ml^−1^ granulocyte macrophage colony-stimulating factor. After incubation overnight, the cells were washed with warm PBS and detached with ice-cold PBS on ice for 30 min. The macrophages were collected, resuspended and plated into 96-, 48- or 24-well plates at a concentration of 10^5^, 5 × 10^5^, or 10^6^ cells per well, respectively, in RPMI 1640 culture media containing 100 units ml^−1^ granulocyte macrophage colony-stimulating factor, and macrophages were further matured for 6 days^52^.

### Macrophage infection assay

*Cryptococcus* strains were cultured in liquid YPD media for 24 h at 25 °C rotating at 20 r.p.m. One millilitre of J774 cells (10^5^ cells per ml) in culture media was plated into each well of a 24-well tissue-culture-treated plate 24 h before infection and kept at 37 °C and 5% CO_2_ humidified atmosphere. One hour before infection, J774 cells and human primary macrophages were switched to DMEM or RPMI 1640, respectively, containing 2 mM glutamine, 100 μg ml^−1^ streptomycin and 100 units ml^−1^ penicillin (assay media), and activated with 150 ng ml^−1^ phorbol 12-myristate 13-acetate. At the same time, *Cryptococcus* cells from 24-h-old liquid cultures were washed three times with PBS, counted in a haemocytometer and 10^6^ yeast cells per 100 μl were opsonized with the monoclonal antibody 18B7 to cryptococcal capsule (0.99 μg ml^−1^, a kind gift from Arturo Casadevall). After pre-incubation, the opsonized yeast cells were directly added to J774 cells or human primary macrophages in fresh assay medium at a ratio of ten yeast cells per macrophage and phagocytosis was allowed to proceed for 2 h at 37 °C and 5% CO_2_ humidified atmosphere. Non-internalized yeast cells were removed by extensive washes with pre-warmed PBS and effectiveness of washing was checked by microscopy^53^,^54^. Infected macrophages were further cultivated at 37 °C in a 5% CO_2_ humidified atmosphere in fresh assay medium until needed^8^,^14^,^25^,^51^,^53^.

### Determination of IPR

For time point *T*=0, 1 ml of DMEM was removed and 200 μl of sterile distilled H_2_O was added into wells to lyse macrophage cells immediately after 2 h infection. After 30 min, the released intracellular yeast were collected. Another 200 μl distilled H_2_O was added to each well to collect the remaining yeast cells. Intracellular yeast were counted using a haemocytometer. For the subsequent time points (*T*=18 h, *T*=24 h and *T*=48 h), intracellular cryptococcal cells were collected and independently counted with a haemocytometer. For each strain tested, the time course was repeated at least three independent times, using different batches of macrophages. The IPR value was calculated by dividing the maximum intracellular yeast number by the initial intracellular yeast number at *T*=0.

For co-infection studies, the fluorescently labelled R265 strain R265_GFP14 (ref. 51) was used to distinguish between low and high proliferating strains. Yeast cells (10^6^ ml^−1^) at a ratio of 1:1 were opsonized with 18B7 for infection of J774 cells and human primary macrophages. For *in vitro* co-incubation, 10^5^ yeast per ml in single or co-cultures with R265_GFP14 at a ratio of 1:1 were incubated in DMEM assay media at 37 °C in a 5% CO_2_ humidified atmosphere for 24 h. Yeast cell numbers were counted on a Nikon Eclipse T*i* using a haemocytometer under fluorescence and bright field.

We confirmed that Trypan Blue stains 100% of the cryptococcal cells in a heat-killed culture, but only ~5% of cells from a standard overnight culture. Compared with a conventional colony counting method, this method was shown to be more sensitive in detecting the clustered yeast population or yeast cells undergoing budding^8^,^14^,^25^,^51^,^53^.

### Mitochondrial staining

*C. gattii* cells were grown overnight at 37 °C in DMEM untreated or under a stress condition (0.7 mM H_2_O_2_, 5 mM NaNO_2_, 0.1 mM CoCl_2_, 3% O_2_, 0.005% SDS, 0.05 mM NaCl, 0.03 J cm^−2^ ultraviolet light, pH 5.7) in a 5% CO_2_ incubator without shaking for 24 h, or isolated from macrophages 24 h after infection. After ultraviolet treatment, yeasts were recovered for 30 min at 37 °C. Growth in YPD media at 25 °C rotating at 20 r.p.m., in YPD media at 37 °C not rotating and assay medium at 37 °C not rotating were also included as controls. For time-course experiments, cells were recovered from macrophages and stained after 0, 2, 6, 12, 18 and 24 h. The cells were harvested, washed with PBS twice and re-suspended in PBS containing the Mito-Tracker Red CMXRos (Invitrogen) at a final concentration of 20 nM. Cells were incubated for 15 min at 37 °C. After staining, cells were washed in triplicate and re-suspended in PBS. For each condition, more than 100 yeast cells per replicate for each of the strains tested were chosen randomly and analysed. For quantifying different mitochondrial morphologies, images were collected using a Zeiss Axiovert 135 TV microscope with a × 100 oil immersion Plan-Neofluar objective. Both fluorescence images and phase-contrast images were collected. Images were captured with identical settings on a QIcam Fast 1394 camera using the QCapture Pro51 version 5.1.1 software. All images were processed identically in ImageJ, and mitochondrial morphologies were analysed and counted blind^14^,^25^.

### Stress treatments

To test *C. gattii* strains for their susceptibility towards a number of stress conditions, several conditions were created to mimic different degrees of hypoxia (0, 0.025, 0.05, 0.075, 0.1, 0.15, 0.3 and 0.6 mM CoCl_2_ and 3% O_2_), oxidative (0, 0.088, 0.175, 0.35, 0.7, 2.1, 4.2 and 9.3 mM H_2_O_2_), nitrosative (0, 0.25, 0.5, 2.5, 5, 10, 15 and 20 mM NaNO_2_), cell membrane (0, 0.005, 0.01, 0.025, 0.05, 0.1, 0.25 and 0.5% SDS), osmotic (0.0001, 0.001, 0.01, 0.025, 0.05, 0.1 and 0.3 mM NaCl), radiation (ultraviolet 254 nm; 0, 0.005, 0.01, 0.02, 0.03, 0.04, 0.06, 0.08 and 0.1 J cm^−2^) and pH (3.6, 4.4, 4.55, 4.56, 4.74, 5.17, 5.28, 5.58, 5.7, 6.5 and 7.5) stress. *Cryptococcus* cells from 24-h-old YPD liquid cultures were washed three times with PBS, counted in a haemocytometer and adjusted to 10^5^ cells per ml in assay media. One millilitre of the yeast suspension was added into each well of a 48-well plate and the stress factor was transferred accordingly. The yeasts were then incubated at 37 °C and 5% CO_2_. To assess the influence of the stresses on cryptococcal survival, serial dilutions were plated after 0 and 24 h, and colony-forming units counted. For ultraviolet treatments, yeasts were recovered for 30 min at 37 °C before plating. Colony-forming units relative to time point 0 and relative to an untreated control were calculated to enable comparison between several *Cryptococcus* strains with different growth rates^50^.

### ROS staining

J774 macrophages, previously prepared according to the infection protocol and treated with 0.5 mM of the ROS inhibitor Apocynin (Sigma) where applicable, were detached and separated by Accutase (PAA) treatment. The cells were incubated with the undiluted proteolytic and collagenolytic enzyme mix for 15 min at 37 °C and then gently dissociated by pipetting to ensure a single cell suspension; cell separation was checked by microscopy. Harvested cells were washed in warm PBS and stained with 2′,7′-dichlorodihydrofluorescein diacetate (Invitrogen) at a concentration of 5 μM in PBS for 37 °C for 45 min. After labelling, macrophages were analysed by flow cytometry (FACSCaliber and CellQuestPro software, BD Biosciences). For analysis, the macrophage population was gated by forward and side scatter, and the percentage of macrophages generating a ROS response measured by negatively gating for dye fluorescence against a population of unlabelled macrophages.

### Live imaging of cryptococcal mitochondria

Time-lapse images for fungal fate analysis were captured on a TE2000 (Nikon) with Digital Sight DS-Qi1MC camera (Nikon), × 60 objective (DIC PLAN APO), using NIS elements AR software (Nikon). Transmitted light images were captured every 2 min and fluorescence images were captured every 10 min for 12 h. Two hundred and eighty-eight and 177 cells for AIg54 and AIg56, respectively, were analysed over four separate experiments. Time-lapse images for fungal fate analysis in the presence of apocynin were captured on a Ti (Nikon) with Neo (Andor), × 60 objective (DIC PLAN APO), using NIS elements AR software (Nikon). Transmitted light images were captured every 2 min and fluorescence images were captured every 10 min for 12 h. After completing the infection protocol, macrophages were treated with either 0.5 mM apocynin or 0.5% dimethyl sulphoxide (control). For strain AIg54 191 control and 129 apocynin-treated macrophages, and for strain AIg56 164 control and 148 apocynin-treated macrophages were analysed over three separate experiments. Three-dimensional imaging of mitochondrial tubularization was acquired with Nikon A1R confocal microscope (Nikon) in resonant scanning mode and with a piezo stage (MadCity Labs) for fast *z*-sectioning. *C. gattii* AIg54 was imaged in PBS containing 0.7 mM H_2_O_2_ to induce tubularization. Seventy-four *z*-sections were captured every 60 s for 40 min.

### *Agrobacterium*-mediated transformation

Strains CBS1930 and R265 were cultured overnight in YPD liquid medium. An *Agrobacterium tumefaciens* strain that carries a plasmid with the *HEM15* gene from *C. neoformans* strain JEC21 fused at its carboxy terminus to GFP was cultured overnight in Luria–Bertani medium supplemented with kanamycin^55^. The fusion protein is under the regulation of a constitutive histone H3 promoter. *HEM15* encodes ferrochelatase, a mitochondrially localized protein that catalyses the final step in the biosynthesis of haem. Each culture was diluted to an optical density of 0.5 at 600 nm. Cultures were mixed, plated onto induction medium agar^56^ and co-cultured for 2 days at room temperature. The cells were then transferred from induction medium to YPD agar containing nourseothicin (100 μg ml^−1^), to select for integration of the T-DNA into the *C. gattii* genome, and cefotaxime (100 μg ml^−1^), to select against the *Agrobacterium* cells. Transformed cells were streaked to isolate single colonies and their fluorescence tested using a Nikon Eclipse 90*i* fluorescence microscope.

### Immunofluorescence of autophagy marker LC3

J774 macrophage-like cells were seeded onto 13 mm glass coverslips at a density of 1 × 10^5^ cells. Macrophages were then incubated at 37 °C and 5% CO_2_ 24 h before infection. *C. gattii* strains (CDCF2932, ENV152, CBS8684, CBS7750, CBS1930, CBS7229, CDCR271 and R265) were cultured and J774 cells infected as previously described for the macrophage infection assay. After 2 h of co-incubation, extracellular yeast were removed by extensive PBS washes and, if appropriate, cells were further incubated in assay media at 37 °C and 5% CO_2_. Cells were fixed with 4% PFA for 10 min at either 2 h (*T*=0) or 20 h (*T*=18) after infection. Coverslips were washed with PBS before treatment with 50 mM NH_4_Cl for 10 min and cells were permeabilized in 0.1% Triton X-100 for 4 min. Coverslips were blocked with 5% goat serum and 1% BSA in PBS for 1 h and incubated with 0.25 μg ml^−1^ rabbit polyclonal anti-LC3A/B antibody (Cell Signaling) for 30 min, followed by 20 min incubation with 3 μg ml^−1^ goat anti-Rabbit IgG-TRITC (Sigma). Coverslips were then mounted in Mowiol (Calbiochem) with p-phenylenediamine antifade agent. Images were captured using a Nikon eclipse Ti-inverted epifluorescence microscope and NIS elements AR 3.2 software (Nikon) with a × 60 objective (Plan Apo VC 1.4 NA DIC N2) and camera (Qimaging QICAM-B Mono). Images were analysed using ImageJ software (National Institutes of Health).

### Hog1 regulation in *C. gattii*

*C. gattii* was grown at 25 °C in YPD media for 24 h. Cells were diluted 1:100 in fresh media and grown to exponential phase at 25 °C. Cultures were diluted 1:1 with YPD, YPD supplemented with 2 M NaCl (final concentration 1 M) or YPD supplemented with 2 mM hydrogen peroxide (final concentration 1 mM) and incubated for an additional 30 min. Cells were harvested by centrifugation and immediately flash-frozen in liquid nitrogen. Pellets were washed in protein extraction buffer (50 mM HEPES pH 7.5, 150 mM NaCl, 5 mM EDTA, 1% NP40) supplemented with Roche complete proteinase cocktail. Samples were lysed by vortexing with glass beads (20 × 30 s). Lysates were cleared by centrifugation (13,000 r.p.m., 1 min) and protein concentration estimated by Bradford assay with a BSA standard curve. Proteins (15 μg) were separated by SDS–PAGE on 15% SDS gels. Proteins were transferred onto polyvinylidene difluoride membranes at 30 V for 2 h and the membrane blocked with 5% BSA in PBS-T for 1 h. Hog1 phosphorylation was detected with a 1:2,000 dilution of phospho-P38 (Thr180/Tyr182) rabbit monoclonal antibody (Cell Signaling) in PBS-T with 5% BSA. Membranes were incubated overnight at 4 °C. For detection, an anti-rabbit IgG-HRP-conjugated antibody was used at a 1:2,000 dilution in PBS-T, 5% BSA for 1 h at room temperature. To detect total Hog1 levels, membrane were stripped, blocked in 10% milk in PBS-T and re-probed with the Sc-9079 Hog1 antibody (Santa Cruz) in a 1:2,000 dilution in 5% milk in PBS-T for 2 h at room temperature. For detection, an anti-rabbit IgG-HRP-conjugated antibody was used in a 1:2,000 dilution in PBS-T for 1 h at room temperature. Membranes were washed in PBS-T and signals detected using an enhanced chemiluminescence western blotting kit as per the manufacturer’s instructions^35^.

### Statistical analysis

Data were analysed using SPSS version 17 and GraphPad Prism 6. Continuous data sets were tested for normal distribution using the Shapiro–Wilks test and for homogeneity of variance using the Levene Statistics, and if data sets fit the requirements, subjected to parametric analysis by *t*-test or analysis of variance with *post-hoc* Tukey test. If data sets were not normally distributed or failed to show homogeneity of variance, the non-parametric Mann-Whitney *U*-test or Kruskal–Wallis test was applied to test for statistically significant differences. Categorical data was pooled and analysed by Fisher’s exact test for low number of data points and *χ*^2^-test for larger data sets. *P*-values <0.05 after adjusting for multiplicity were considered statistically significant. Experiments were performed at least three times. Exact details on experimental repeats and number of yeast scored can be found in Supplementary Tables 2–6. Data were not excluded from analysis unless obvious operator error occurred (for example, failure to stain and contamination). Scoring experiments were performed in a blind manner. For *in vitro* studies and infection studies, fungal strains were randomly distributed in different culture well plate positions.

## Additional information

**How to cite this article:** Voelz, K. *et al.* ‘Division of labour’ in response to host oxidative burst drives a fatal *Cryptococcus gattii* outbreak. *Nat. Commun.* 5:5194 doi: 10.1038/ncomms6194 (2014).

## Supplementary Material

Supplementary Figures, Tables and References Supplementary Figures 1-7, Supplementary Tables 1-6 and Supplementary References

Supplementary Movie 1 Mitochondrial tubularisation of yeasts. This was observed by confocal live cell imaging in the outbreak strain AIg54. Images were generated by projecting 73 z-planes in a single plane with each z-plane given an individual colour. The globular morphology therefore gives a multi-colour unsaturated object due to the combination of many different colours and intensities over the z-planes, while tubular mitochondria in either the x- or y-plane give a more uniform, saturated colour. Scale bar 1 μm).

## Figures and Tables

**Table 1:** *Cryptococcus* strains and details about serotype, genotype, AFLP type and source.

**Species and strains**	**Sero-type**	**Geno-type**	**AFLP**	**Source**
*C. gattii* CBS10089	B	VGII	6	Clinical isolate from Greece
*C. gattii* CBS10090	B	VGII	6	Clinical isolate from Greece
*C. gattii* CBS10101	C	VGIV	7	Isolate from King Cheeta, South Africa
*C. gattii* CBS10485	B	VGIIa	6	Clinical isolate from Danish tourist who had visited Vancouver Island
*C. gattii* CBS6955	C	VGIII	5	Clinical isolate, California, USA
*C. gattii* CBS6993	B	VGIII	5	Clinical isolate, cerebrospinal fluid, California, USA
*C. gattii* CDCF3016	B	VGIIa	6	Isolate from dead wild Dall’s porpoise from the shores of an island close to Vancouver Island, Canada
*C. gattii* NIH312xCBS10090 progeny 5	N/A	N/A	N/A	Progeny from cross between *C. gattii* strains NIH312 and CBS10090
*C. gattii* EJB18	B	VGIIc	6	Clinical isolate, Oregon, USA
*C. gattii* EJB52	B	VGIIc	6	Clinical isolate, Oregon, USA
*C. gattii* ICB180	B	VGII	6	Environmental isolate, Eucalyptus tree, Brazil
*C. gattii* ICB184	B	VGII	6	Environmental isolate, tree, Brazil
*C. gattii* LA362	B	VGII	6	Isolate from parrot litter, Jaboticabal, Brazil
*C. gattii* LMM265	B	VGII	6	Clinical isolate, Brazil
*C. gattii* WM276	B	VGI	4	Environmental isolate, Australia
*C. gattii* NIH312	C	VGIII	5	Clinical isolate
*C. gattii* R265_GFP14	B	VGIIa	6	GFP expressing *C. gattii* R265
*C. gattii* R265	B	VGIIa	6	Clinical isolate from Duncan, British Columbia, Canada
*C. gattii* CDCR271	B	VGIIa	6	Clinical isolate, immunocompetent patient, Kelowna, British Columbia, Canada
*C. gattii* CDCF2932	B	VGIIa	6	Clinical isolate, immunocompetent male, British Columbia, Canada
*C. gattii* ENV152	B	VGIIa	6	Environmental isolate, Alder tree, Vancouver Island, Canada
*C. gattii* CBS8684	B	VGII	6	Environmental isolate, wasp nest, Uruguay
*C. gattii* CBS7750	B	VGIIa	6	Environmental isolate, *E. camaldulensis*, USA
*C. gattii* CBS1930	B	VGIIb	6	Sick goat, Aruba
*C. gattii* CBS7229	B	VGI	4	Clinical isolate, meningitis, China
*C. gattii* AIg54	B	VGIIa	6	HEM15-GFP tagged strain derived from R265
*C. gattii* AIg56	B	VGII	6	HEM15-GFP tagged strain derived from CBS1930
*C. neoformans* ATCC90112	A	VNI	1	Clinical isolate, cerebrospinal fluid, USA
*C. neoformans* CBS8336	A	VNI	1	Environmental isolate, wood of Cassia tree, Brazil
*C. neoformans* H99	A	VNI	1	Clinical isolate, USA
*C. neoformans* BD5	D	VNIV	2	Clinical isolate, AIDS patient, France
*C. neoformans* CBS6995	D	VNIV	2	Clinical isolate, USA
*C. neoformans* JEC21	D	VNIV	2	Derived from a clinical isolate, AIDS patient, USA
*C. neoformans* A1-84-14	A	VNI	1	Environmental isolate, pigeon guano, California, USA
*C. neoformans* A5-35-17	A	VNI	1	Environmental isolate, pigeon guano, North Carolina, USA
*C. neoformans* Tu406-1	A	VNII	1A	Environmental isolate, Mopane tree bark, Botswana
*C. neoformans* A1-38-2	A	VNI	1	Environmental isolate, pigeon guano, North Carolina, USA
*C. neoformans* Tu369-2	A	VNII	1A	Environmental isolate, Mopane tree bark, Botswana
*C. neoformans* A4-34-6	A	VNI	1	Environmental isolate, pigeon guano, North Carolina, USA
*C. neoformans* A7-35-23	A	VNI	1	Environmental isolate, soil, North Carolina, USA
*C. neoformans* A1-35-8	A	VNI	1	Environmental isolate, pigeon guano, North Carolina, USA

AFLP, amplified fragment length polymorphism; GFP, green fluorescent protein.

Strain references can be found in Supplementary Table 1.

**Table 2:** Proliferation rates (IPR) and percentage of yeast with tubular mitochondria (tubularization) after encounter of the intracellular macrophage niche for *C. gattii* and *C. neoformans* strains with different genotypes.

**Strain**	**IPR**	**Tubularization (%)**	**Genotype**
*C. gattii*			
ICB180	0.7±0.1	9.2±1.0	VGII
CBS10089	0.8±0.1	13.8±5.4	VGII
ICB184	0.8±0.1	20.5±7.4	VGII
CBS6955	0.9±0.1	7.5±3.5	VGIII
NIH312	0.9±0.1	8.6±3.6	VGIII
CBS8684	1.0±0.2	6.2±1.8	VGII
CBS7229	1.0±0.1	20.7±1.0	VGI
WM276	1.0±0.1	13.8±2.0	VGI
NIH312xCBS10090 Progeny 5	1.1±0.2	6.7±1.3	VGIII x VGII
CBS1930	1.1±0.1	23.9±9.4	VGIIb
CBS10101	1.2±0.1	20.4±7.7	VGIV
EJB52	1.4±0.1	17.5±7.4	VGIIc
CBS6993	1.6±0.2	29.4±12.3	VGIII
LA362	1.6±0.3	30.7±4.0	VGII
CDCF3016	1.7±0.1	31.1±6.3	VGIIa
EJB18	1.7±0.1	43.5±19.5	VGIIc
R265	1.8±0.1	58.4±17.6	VGIIa
CBS10090	1.9±0.2	46.9±10.4	VGII
CBS10485	1.9±0.1	37.9±8.9	VGIIa
R265_GFP14	2.0±0.1	51.2±8.1	VGIIa
CDCR271	2.1±0.4	48.4±5.7	VGIIa
CDCF2932	2.2±0.1	52.8±13.4	VGIIa
ENV152	2.3±0.2	44.5±6.1	VGIIa
LMM265	2.3±0.2	34.6±14.1	VGII
			
*C. neoformans*			
CBS8336*	1.2±0.4	53.3±7.1	VNI
A5_35_17	1.3±0.1	65.1±10.4	VNI
CBS6995*	1.3±0.3	55.2±5.3	VNIV
H99*	1.4±0.2	37.5±4.9	VNI
A4_34_6	1.8±0.2	69.0±10.6	VNI
Tu_369_1	1.9±0.1	23.2±12.3	VNII
BD5*	1.9±0.3	31.0±5.9	VNIV
A1_38_2	2.0±0.2	54.5±13.0	VNI
JEC21*	2.1±0.2	39.9±7.5	VNIV
A1_35_8	2.2±0.2	61.1±3.8	VNI
A7_35_23	2.2±0.1	13.8±12.4	VNI
Tu_406_1	2.6±0.3	37.0±10.0	VNII
ATCC90112*	2.9±0.2	32.3±9.2	VNI
A1_84_14	3.4±0.2	44.8±5.6	VNI

IPR, intracellular proliferation rate.

IPR and tubularization data is presented as mean averages with s.e.m. (values indicated by * were taken from ref. 57). Categorical tubularization data was obtained from three independent experimental repeats examining between 325 and 3,598 yeast. Outbreak strains are highlighted in red.

**Table 3:** Intracellular yeast fate analysis identifies yeast with tubular mitochondria as resistant and non-proliferative cells.

	**Mitochondrial morphology**				**Fisher’s exact test**
	**Tubular**		**Non-tubular**		
	**Count**	**%**	**Count**	**%**	
*AIg54*					
Total	69	24.0	219	76.0	
Killed	0	0	49	22.4	*P*<0.0001
Proliferating	1	1.4	35	16.0	*P*=0.0006
Quiescent	68	98.6	134	61.2	*P*<0.0001
					
*AIg56*					
Total	17	9.9	154	90.1	
Killed	0	0	2	1.3	*P*=1.0
Proliferating	10	58.8	80	51.9	*P*=0.62
Quiescent	7	41.2	74	48.1	*P*=0.62

The fate of individual intracellular yeasts and their respective mitochondrial phenotype was scored from time-lapse images over 12 h (730 min). Fewer intracellular yeast were killed when tubular mitochondrial morphology has developed (Fisher’s exact test, *P*<0.0001). Intracellular yeast proliferation was more often observed in yeasts from the outbreak strain AIg54 not presenting with tubular mitochondria (Fisher’s exact test, *P*=0.0006). Intracellular AIg54 cells with tubular mitochondrial morphology were more often quiescent than intracellular AIg56 cells with tubular mitochondria (Fisher’s exact test, *P*<0.0001). Data were obtained from the outbreak strain AIg54 and non-outbreak strain AIg56 with HEM15-GFP-tagged mitochondria in four independent experimental repeats examining 288 and 173 yeasts, respectively, and data analysed using Fisher’s exact test.

**Table 4:** Outbreak and non-outbreak strains can co-infect macrophages.

**a**		
**Strains**	**Total (%)**	
	**Average**	**s.e.m.**
*Percentage phagocytosis*		
R265_GFP14+CBS8684, *n*=4	36.4	1.4
R265_GFP14+CBS7750, *n*=3	38.6	3.4
R265_GFP14+CBS7229, *n*=4	35.9	3.4
R265_GFP14+CBS1930, *n*=4	28.1	2.6

**b**						
**Strain**	**R265_GFP14**		**Non-outbreak strain**		**Co-infected**	
	**Average**	**s.e.m.**	**Average**	**s.e.m.**	**Average**	**s.e.m.**
*Co-infection percentage phagocytosis (% of total above)*						
CBS8684	92.2	2.0	14.2	1.3	8.2	0.5
CBS7750	86.6	4.3	22.7	4.2	9.4	0.8
CBS7229	74.9	2.5	46.0	1.7	21.9	3.0
CBS1930	91.2	2.5	18.7	1.4	9.7	1.2

Phagocytosis of outbreak strain R265_GFP14 and non-outbreak strains during co-infection was analysed and percentage phagocytosis of yeast cells after 2 h of co-infection by J774 macrophages are presented (*n*>3). The total uptake (a) and contribution of single and co-infection to total uptake (b) are shown. Data for assessment of phagocytosis were obtained from at least three independent experimental repeats.
