# Supplementary material for: ‘Division of labour’ in response to host oxidative burst drives a fatal Cryptococcus gattii outbreak
Source: Nat Commun. 2014 Oct 17;5:5194. doi: 10.1038/ncomms6194 (PMC4208095; doi:10.1038/ncomms6194)
Supplement: Supplementary Figures, Tables and References — Supplementary Figures 1-7, Supplementary Tables 1-6 and Supplementary References [file ncomms6194-s1.pdf]

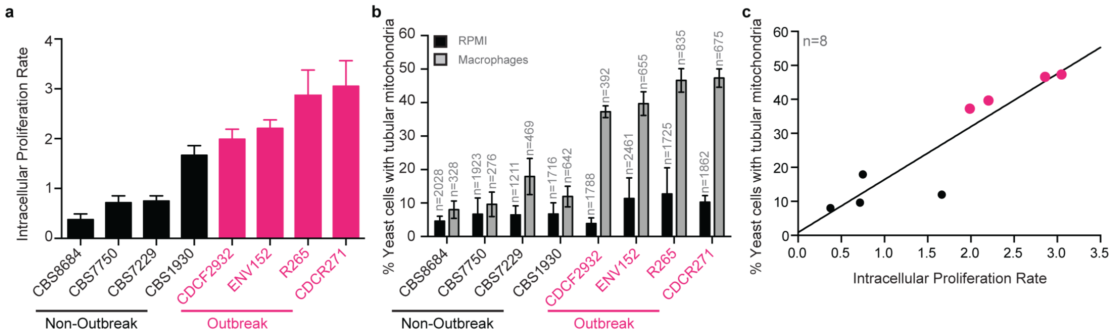

Supplementary Figure 1 **Intracellular yeast cell fate in primary human macrophages from peripheral blood. a)** Intracellular proliferation of four non-outbreak and four outbreak strains after phagocytosis by primary human macrophages isolated from peripheral blood. Data were collected from six independent experiments, presented as mean averages with SEM. **b)** Mitochondrial morphology of four non-outbreak and four outbreak strains was assessed after phagocytosis by primary human macrophages isolated from peripheral blood and under control conditions (RPMI growth media). Categorical data were collected from three independent repeats examining mitochondrial morphology in 276-2,461 yeast cells. **c)** The formation of tubular mitochondria in response to encounter of the intracellular niche positively correlates with the ability to proliferate within macrophages (Linear regression, Pearson Correlation: R^2=0.848; p=0.001). Data were obtained from results presented in Supplementary Fig. 1a, b.

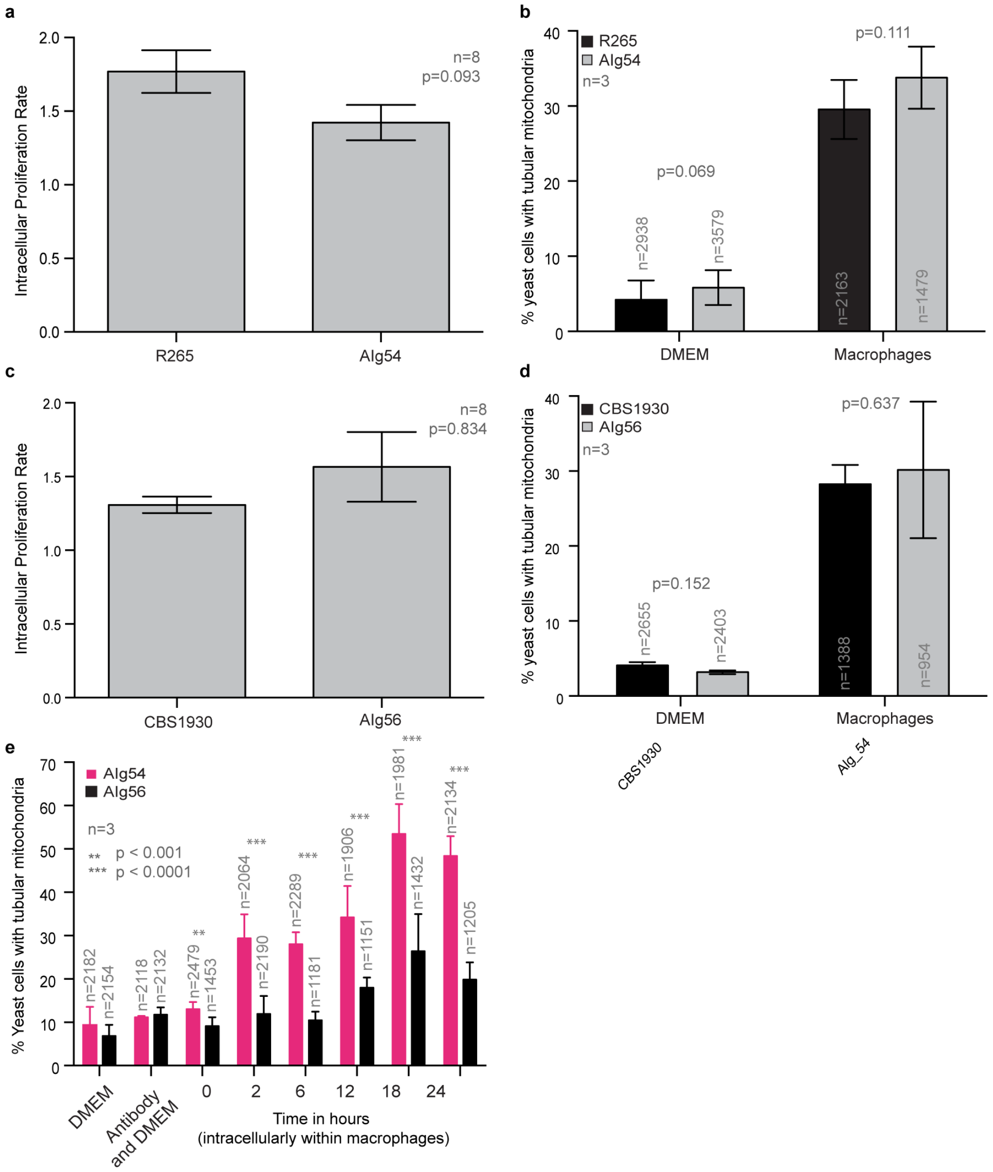

Supplementary Figure 2 **Analysis of transgenic strains expressing GFP.** The outbreak and the non-outbreak cryptococcal HEM15-GFP strains AIg54 and AIg56, respectively, with genetically encoded GFP-tagged mitochondria do not show altered behaviour in a macrophage model. **a and c)** Intracellular proliferation of the parental strains R265 and CBS1930 and the respective mutant HEM15-GFP strains AIg54 and AIg56 in J774 cells is not significantly different. Data were collected from eight independent experiments, presented as mean averages with SEM. Data was analysed by Mann-Whitney U-test. **b and d)** Mitochondrial tubularisation of the parental strains R265 and CBS1930 and the respective HEM15-GFP strains AIg54 and AIg56 under control conditions or encounter of the intracellular macrophage niche in J774 cells is not significantly different. Categorical data were collected from three independent repeats examining mitochondrial morphology in 1,479-3,579 yeast cells and analysed with Fisher's Exact Test. **e)** Mitochondrial tubularisation of both strains AIg54 and AIg56 was observed with MitoTracker CMXRos in a macrophage infection time course experiment. Fast initiation of tubularisation was revealed in the outbreak strain AIg54 but a delayed and much lower tubularisation response in the non-outbreak strain AIg56. Categorical data (tubular versus non-tubular mitochondria) from four independent experiments observing between 1,151 and 2,479 yeasts were analysed by Fisher's Exact Test.

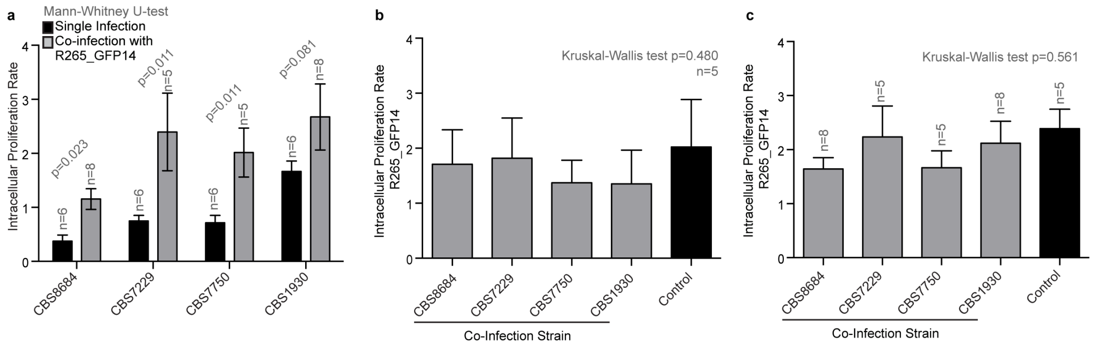

Supplementary Figure 3 **Co-infection studies of outbreak and non-outbreak strains within macrophages. a)** Co-infection of non-outbreak strains with R265_GFP14 also increases intracellular proliferation rates of non-outbreak strains CBS8684, CBS7229 and CBS7750 (p=0.023, p=0.011, p=0.011, respectively) in primary human monocyte-derived macrophages. IPR data for co-infections was obtained in parallel with IPR data for Supplementary Figure 1 and IPR data for single infections is the same in both figures. **b and c)** The proliferative potential of R265_GFP14 after phagocytosis by J774 (b) or primary human macrophages isolated from peripheral blood (c) was not altered (p=0.480 and p=0.562, respectively). Intracellular proliferation data were obtained from at least five independent experimental repeats and presented as mean averages with SEM and analysed by Mann-Whitney U-test or Kruskal-Wallis test.

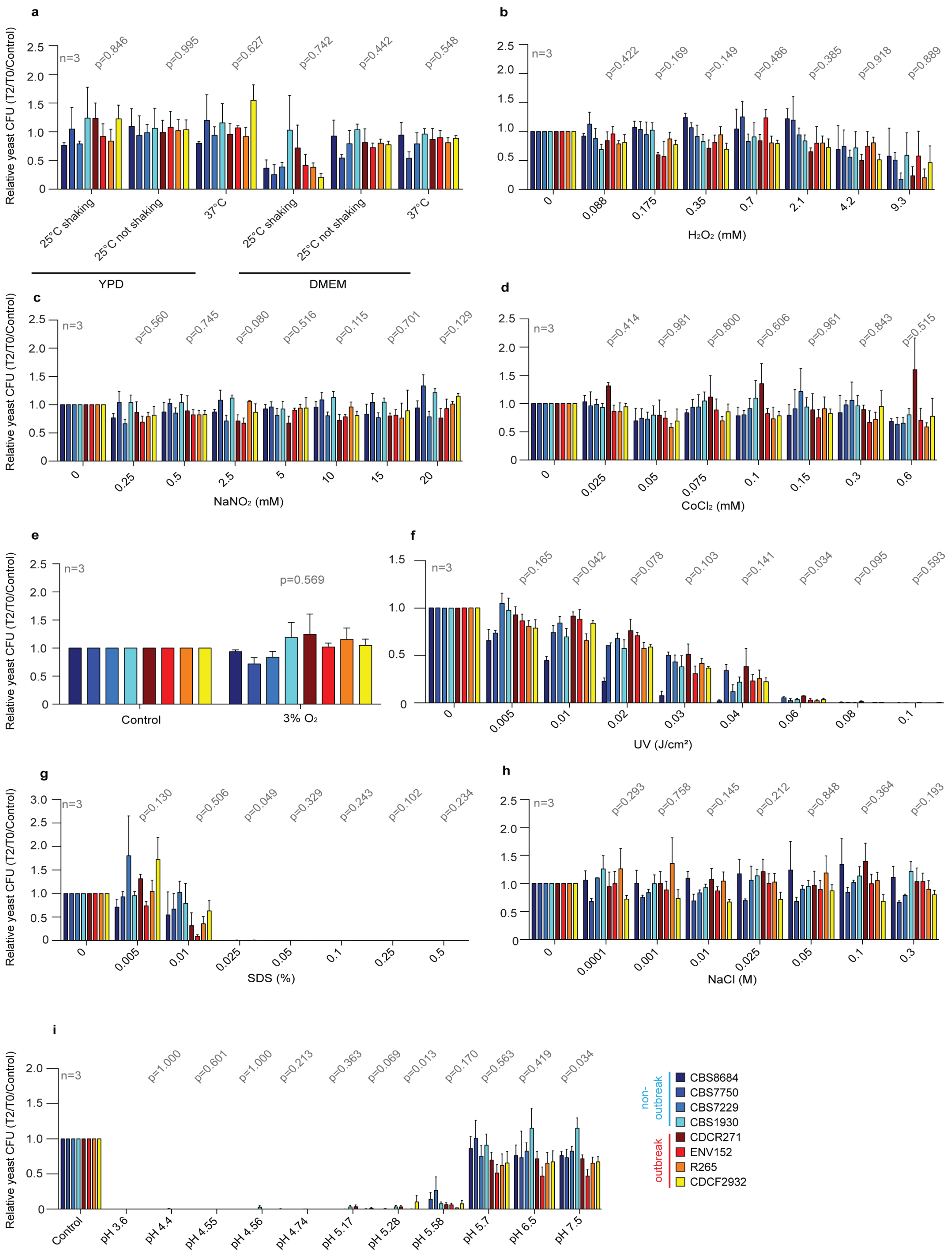

Supplementary Figure 4 ***C. gattii* survival under stress conditions.** Four outbreak strains and four non-outbreak strains were tested for their survival under oxidative (H_2O_2), nitrosative ($NaNO_2$), low oxygen ($CoCl_2$, 3% O_2), UV, cell wall (SDS, NaCl) and acid (pH) stress **(a-i)**. No trends towards improved or reduced survival of the outbreak population compared to the non-outbreak population after two hours of exposure to the stresses was observed. Data were obtained from three independent experimental repeats and presented as mean averages with SEM and analysed by Mann-Whitney U-test.

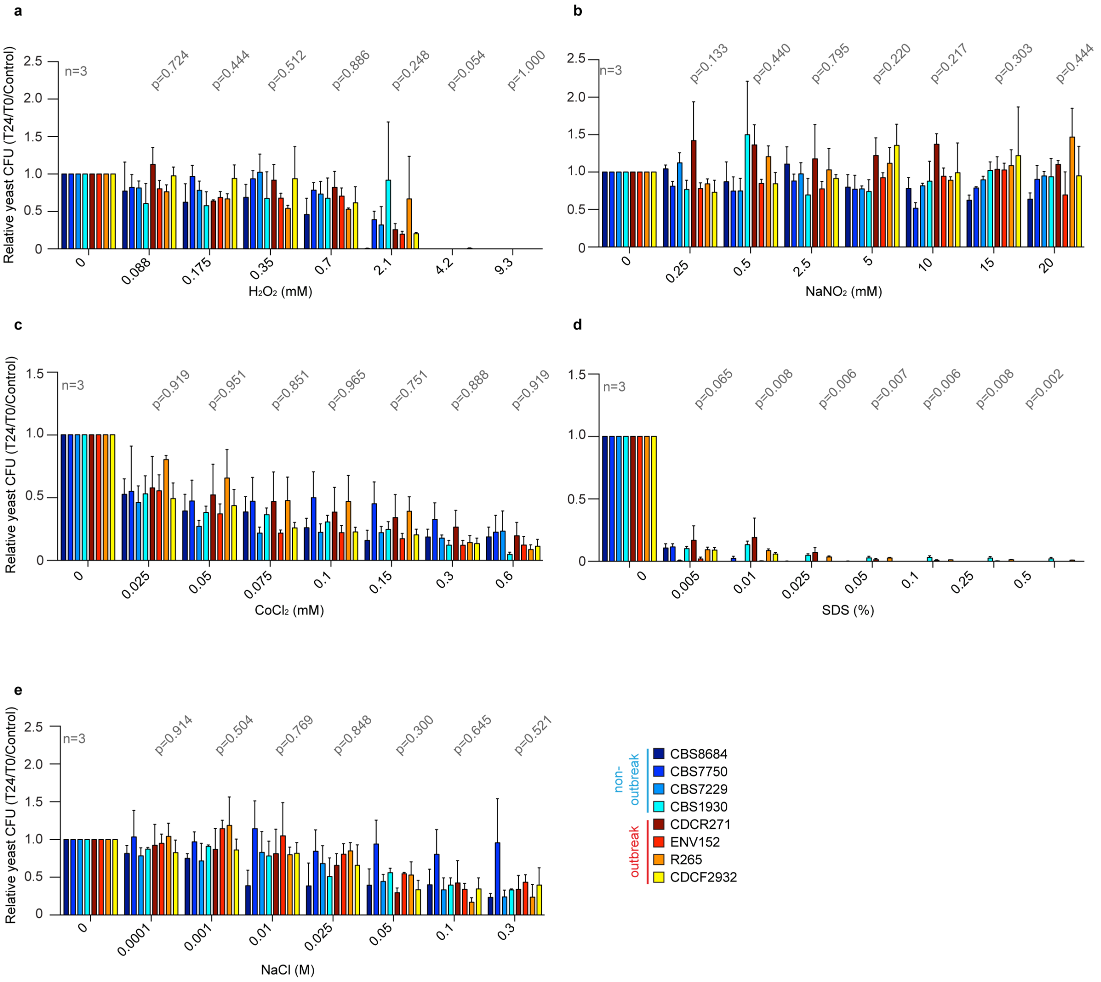

Supplementary Figure 5 ***C. gattii*** **growth under stress conditions.** Four outbreak strains and four non-outbreak strains were tested for their growth under oxidative (H_2O_2), nitrosative ($NaNO_2$), low oxygen ($CoCl_2$, 3% O_2) and cell wall (SDS, NaCl) stress **(a-e)**. No trend towards improved or reduced survival of the outbreak population compared to the non-outbreak population after 24 hours of exposure to the stresses was observed. Data were obtained from three independent experimental repeats and presented as mean averages with SEM and analysed by Mann-Whitney U-test.

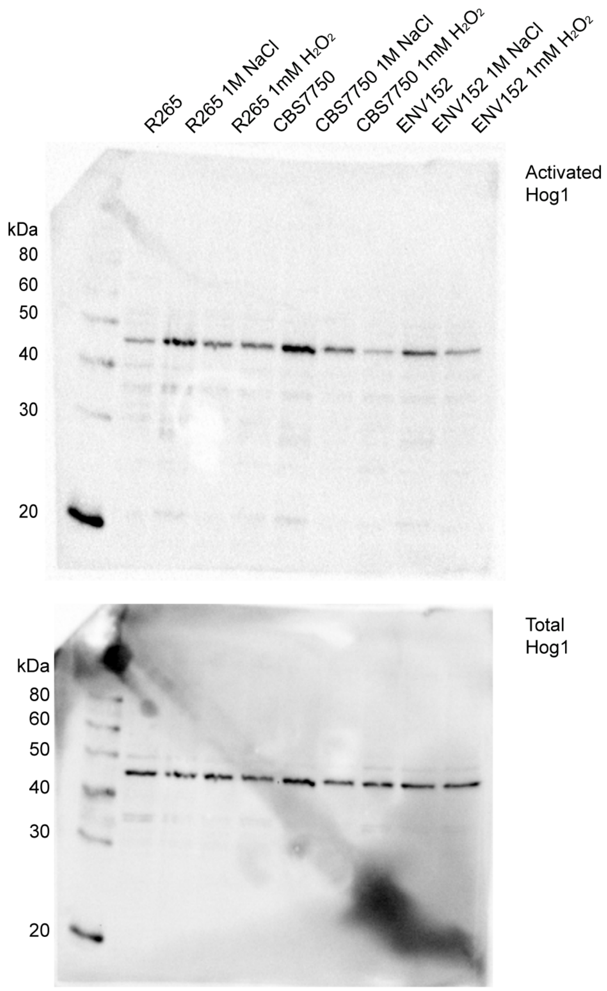

Supplementary Figure 6 **Hog1 phosphorylation is not differentially regulated in response to oxidative or osmotic stress in clinical or environmental strains of *C. gattii.*** Exponential phase cells were stressed for 30 minutes, flash-frozen and crude protein extracted. Protein (15 µg) was separated by SDS-PAGE and phosphorylated and total Hog1 levels measured by western blot. As previously reported, Hog1 phosphorylation is induced by osmotic shock, but activation of this kinase was similar for all three strains tested in response to NaCl or reactive oxygen species.

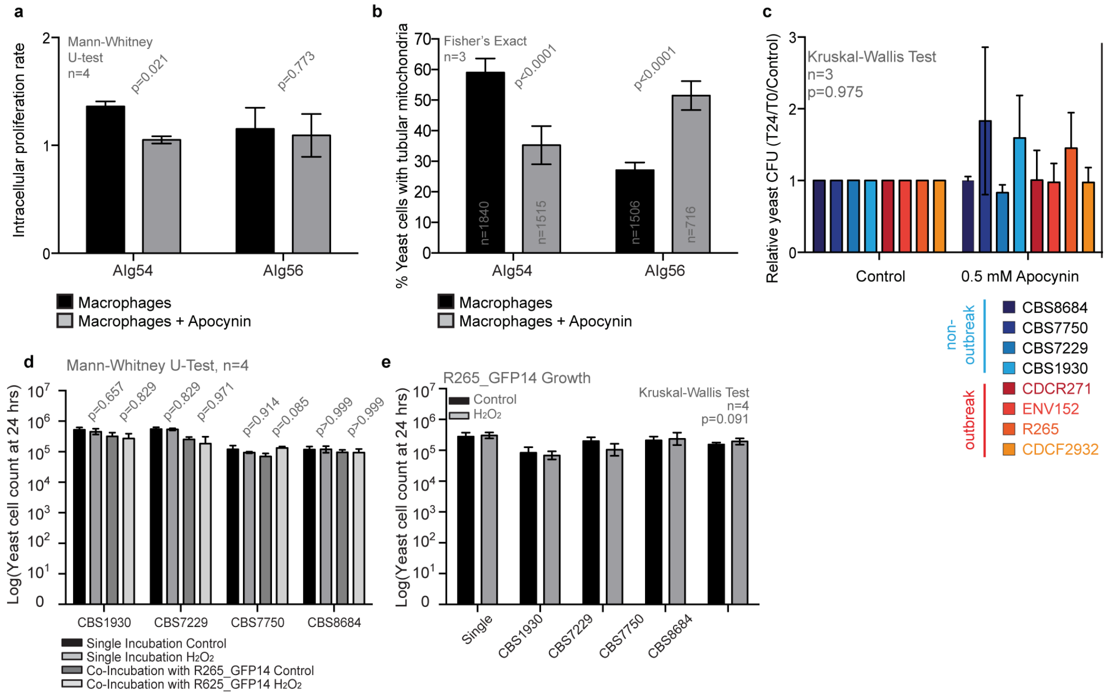

Supplementary Figure 7 **Apocynin manipulation of ROS**. **a and b)** Inhibition of ROS reduced the proportion of intracellular yeasts presenting with tubular mitochondria ($p<0.0001$) in the outbreak strain AIg54 and significantly reduced the strain's ability to proliferate intracellularly ($p=0.021$). Intracellular proliferation was not significantly changed in the non-outbreak strain AIg56 ($p=0.773$) whilst mitochondrial tubularisation was significantly increased ($p<0.0001$). Data were obtained from four individual experiments determining intracellular proliferation rate, presented as mean averages with SEM and analysed by Mann-Whitney U-test. Categorical data (tubular versus non-tubular mitochondria) from three independent experiments observing between 716 and 1,840 yeasts were analysed by Fisher's Exact Test. **c)** Treatment with 0.5 mM apocynin did not significantly alter the growth of any strains tested. Data were obtained from three independent experimental repeats and presented as mean averages with SEM and analysed by Kruskal-Wallis test. **d and e)** Co-cultures of non-outbreak strains with the outbreak strain R265_GFP14 grown *in vitro* in the presence of 0.7 mM H_20_2 did not increase proliferation of non-outbreak strains or alter proliferation in the co-incubation strain R265_GFP14, suggesting that ROS-induced mitochondrial tubularisation induces a pathogen response that protects cryptococci from their macrophage host, rather than conferring a direct resistance to oxidative damage per se. Data were obtained from four independent experimental repeats and presented as mean averages with SEM and analysed by Mann-Whitney U-test or Kruskal-Wallis Test.

Supplementary Table 1: References for strains used in this study

**Species and Strains**	**Reference**
C. gattii CBS10089	Velegraki *et al.* 2001[1]
C. gattii CBS10090	Velegraki *et al.* 2001[1]
C. gattii CBS10101	Bolton *et al.* 1999[2]
C. gattii CBS10485	Lindberg *et al.* 2007[3]
C. gattii CBS6955	Kwon-Chung 1976[4]
C. gattii CBS6993	Boekhout *et al.* 1997[5]
C. gattii CDCF3016	Kidd *et al.* 2004[6]
C. gattii NIH312xCBS10090 progeny 5	Voelz *et al.* 2013[7]
C. gattii EJB18	Byrnes *et al.* 2010[8]
C. gattii EJB52	Byrnes *et al.* 2010[8]
C. gattii ICB180	Hagen *et al.* 2012[9]
C. gattii ICB184	Pereira *et al.* 2009[10]
C. gattii LA362	Meyer *et al.* 2003[11]
C. gattii LMM265	Trilles *et al.* 2008[12]
C. gattii WM276	Chaturvedi *et al.* 2005[13]
C. gattii NIH312	Schmeding *et al.* 1981[14]
C. gattii R265_GFP14	Voelz *et al.* 2010[15]
C. gattii R265	Kidd *et al.* 2004[6]
C. gattii CDCR271	Kidd *et al.* 2004[6]
C. gattii CDCF2932	Kidd *et al.* 2004[6]
C. gattii ENV152	Kidd *et al.* 2004[6]
C. gattii CBS8684	Fraser *et al.* 2005[16]
C. gattii CBS7750	Boekhout *et al.* 1997[5]
C. gattii CBS1930	Boekhout *et al.* 1997[5]
C. gattii CBS7229	Boekhout *et al.* 1997[5]
C. gattii AIg54	this study
C. gattii AIg56	this study
C. neoformans ATCC90112	Espinel-Ingroff *et al.* 1992[17]
C. neoformans CBS8336	Lazera *et al.* 1996[18]
C. neoformans H99	Perfect *et al.* 2001[19]
C. neoformans BD5	Boekhout *et al. 2001*[20]
C. neoformans CBS6995	Boekhout *et al.* 1997[5]
C. neoformans JEC21	Kwon-Chung, Edman & Wickes 1992[21]
C. neoformans A1-84-14	Litvintseva & Mitchell 2009[22]
C. neoformans A5-35-17	Litvintseva & Mitchell 2009[22]
C. neoformans Tu406-1	Litvintseva *et al.* 2011[23]
C. neoformans A1-38-2	Litvintseva & Mitchell 2009[22]
C. neoformans Tu369-2	Litvintseva *et al.* 2011[23]
C. neoformans A4-34-6	Litvintseva & Mitchell 2009[22]
C. neoformans A7-35-23	Litvintseva & Mitchell 2009[22]
C. neoformans A1-35-8	Litvintseva & Mitchell 2009[22]

Supplementary Table 2: Experimental repeats (n) and number of yeast cells observed for data presented in Figure 1.

**Strain**	**IPR (n)**	**Tubularisation (n)**	**(yeast scored)**
C. gattii			
ICB180	4	3	759
CBS10089	4	3	1261
ICB184	6	3	444
CBS6955	6	3	324
NIH312	7	3	493
CBS8684	8	3	471
CBS7229	4	3	979
WM276	5	3	859
NIH312xCBS10090 Progeny 5	7	3	420
CBS1930	8	3	504
CBS10101	4	3	490
EJB52	5	3	568
CBS6993	6	3	871
LA362	4	3	1176
CDCF3016	7	3	1117
EJB18	7	3	780
R265	5	3	1649
CBS10090	7	3	1858
CBS10485	7	3	1443
R265_GFP14	7	3	1017
CDCR271	5	3	1024
CDCF2932	5	3	958
ENV152	7	3	606
LMM265	6	3	1375
C. neoformans			
CBS8336	3	3	961
A5_35_17	8	3	1410
CBS6995	3	3	2185
H99	3	3	2319
A4_34_6	8	3	2696
Tu_369_1	8	3	2435
BD5	3	3	2029
A1_38_2	8	3	2596

JEC21	3	3	3589
A1_35_8	8	3	2293
A7_35_23	8	3	3150
Tu_406_1	8	3	2691
ATCC90112	3	3	2325
A1_84_14	8	3	2499

Supplementary Table 3: Experimental repeats (n) and number of yeast cells observed for data presented in Figure 2.

**Time**	**CDCF2932**		**CBS8684**	
	**n**	**yeast scored**	**n**	**yeast scored**
DMEM	3	4059	3	2359
AB and DMEM	3	3496	3	4114
0	4	1143	4	2071
2	4	894	4	1226
6	4	1415	3	1806
12	4	1594	4	1579
18	4	1677	4	2497
24	3	1293	3	1470

Supplementary Table 4: Mean averages ± SEM, experimental repeats (n) and number of yeast cells observed for data presented in Figure 5 a-i. **a)** IPR; **b)** Mitochondrial tubularisation.

a)

**Strain**	**IPR Mean±SEM**	**n**
CBS8684	0.9±0.2	17
CBS7229	1.0±0.1	13
CBS7750	1.0±0.1	9
CBS1930	1.2±0.1	17
CDCR271	1.9±0.2	14
R265	1.9±0.1	14
ENV152	2.1±0.2	16
CDCF2932	2.3±0.1	14

b)

	**Tubularisation**											
	**CBS8684**			**CBS7229**			**CBS7750**			**CB1930**		
	**Mean± SEM**	**n**	**yeast scored**	**Mean± SEM**	**n**	**yeast scored**	**Mean± SEM**	**n**	**yeast scored**	**Mean± SEM**	**n**	**yeast scored**
Macrophages	2.9±0.7	8	2507	1.0±1.0	4	691	8.1±2.9	5	848	11.8±1.2	3	646
H_2O_2	2.3±0.2	3	1551	2.8±0.2	3	502	8.7±0.6	3	782	9.1±2.4	3	567
$NaNO_2$	10.2±3.9	4	1345	5.3±1.6	4	1514	7.3±1.0	3	1069	6.4±1.6	4	947
$CoCl_2$	4.0±1.4	4	1794	6.9±1.0	4	1318	8.0±2.0	4	1257	11.8±1.0	4	1120
O_2	5.7±1.2	4	2169	8.9±2.3	4	838	9.8±1.6	4	2271	17.1±4.3	4	1065
UV	3.9±0.1	3	1484	5.7±0.6	3	1290	3.9±0.2	3	1443	9.2±3.6	3	1942
SDS	13.7±4.5	4	2708	13.9±2.8	4	4859	12.2±0.8	4	3255	18.0±4.9	4	2731
NaCl	9.7±0.7	3	1051	22.2±5.4	3	888	24.4±2.8	3	1212	14.4±2.3	3	786
Acid	35.8±1.0	3	5550	31.3±0.8	3	5751	3.5±0.7	3	6810	16.0±3.0	3	5770
	**CDCR271**			**R265**			**ENV152**			**CDCF2932**		
	**Mean± SEM**	**n**	**yeast scored**	**Mean± SEM**	**n**	**yeast scored**	**Mean± SEM**	**n**	**yeast scored**	**Mean± SEM**	**n**	**yeast scored**
Macrophages	21.3±2.5	5	1043	24.5±3.2	5	1267	33.8±6.9	4	1679	43.2±6.0	4	1483
H_2O_2	45.5±13.6	3	594	38.5±7.4	3	590	35.4±10.6	3	873	51.8±9.2	3	820

$NaNO_2$	11.3±4.8	4	1225	14.3±4.0	4	1325	15.0±3.7	4	1410	16.6±4.2	4	1788
$CoCl_2$	23.8±7.2	4	1406	24.2±4.3	4	1485	26.3±6.4	4	1176	25.7±5.9	4	1205
O_2	18.3±1.1	4	1126	16.7±8.4	4	2467	18.8±5.9	3	872	28.0±5.4	4	1115
UV	15.7±0.3	3	1294	20.3±2.6	3	1406	8.2±2.2	3	1736	28.5±2.7	3	1149
SDS	4.2±0.5	3	2163	2.5±0.3	4	3732	3.9±0.5	4	3066	5.8±0.8	4	3477
NaCl	11.7±1.5	4	1085	21.4±3.5	3	878	12.0±2.5	3	889	13.7±3.2	4	1440
Acid	16.2±6.3	3	4851	22.4±9.4	3	3870	18.3±6.2	3	5000	18.7±4.0	3	4903

Supplementary Table 5: Mean averages ± SEM and number of yeast cells observed for data from the four outbreak and four non-outbreak strains presented in Figure 5 j.

	**Tubularisation**			
	**Non-outbreak strains**		**Outbreak strains**	
	**Mean± SEM**	**yeast scored**	**Mean± SEM**	**yeast scored**
YPD 25°C shaking	1.2±0.3	5933	1.0±0.3	5230
YPD 37°C not shaking	2.0±0.5	5234	2.0±0.3	5219
DMEM 37°C not shaking	2.6±0.7	3894	3.0±0.6	3938
Macrophages	6.0±2.5	4692	30.7±4.9	5482
H_2O_2	5.7±1.8	3402	42.8±3.7	3828
$NaNO_2$	8.3±1.4	4875	14.3±1.1	5748
$CoCl_2$	7.7±1.6	5489	25.0±0.6	5272
O_2	10.4±2.4	6343	20.4±2.6	5580
UV	5.7±0.7	6159	18.2±0.6	5585
SDS	14.5±1.2	13553	4.1±2.3	12438
NaCl	17.7±3.4	3937	14.7±2.3	4292
Acid	21.7±7.4	23881	18.9±1.3	18624

Supplementary Table 6: Mean averages ± SEM and number of yeast cells observed for data from the four outbreak and four non-outbreak strains presented in Figure 6c.

	**Tubularisation**			
	**Control**		**Apocynin**	
	**Mean± SEM**	**yeast scored**	**Mean± SEM**	**yeast scored**
**CBS8684**	2.6±1.7	1129	1.4±0.9	996
**CBS7750**	7.0±0.5	1267	8.6±1.1	612
**CBS7229**	11.2±4.4	1494	12.6±3.5	1548
**CBS1930**	27.9±13.2	914	33.1±11.4	669
**CDCR271**	27.3±8.4	1589	15.7±7.0	1258
**ENV152**	34.9±7.5	1528	18.4±7.3	971
**R265**	43.5±8.3	1280	21.6±10.9	1204
**CDCF2932**	24.6±10.5	2209	18.2±5.4	1429

Supplementary References

1 Velegraki, A. *et al.* First report of *Cryptococcus neoformans* var. *gattii* serotype B from Greece. *Med Mycol* **39**, 419-422 (2001).

2 Bolton, L. A. *et al.* Cryptococcosis in captive cheetah (Acinonyx jubatus): two cases. *J S Afr Vet Assoc* **70**, 35-39 (1999).

3 Lindberg, J., Hagen, F., Laursen, A., Stenderup, J. & Boekhout, T. *Cryptococcus gattii* risk for tourists visiting Vancouver Island, Canada. *Emerg Infect Dis* **13**, 178-179 (2007).

4 Kwon-Chung, K. J. A new species of Filobasidiella, the sexual state of *Cryptococcus neoformans* B and C serotypes. *Mycologia* **68**, 943-946 (1976).

5 Boekhout, T. *et al.* Molecular typing of *Cryptococcus neoformans*: taxonomic and epidemiological aspects. *Int J Syst Bacteriol* **47**, 432-442 (1997).

6 Kidd, S. E. *et al.* A rare genotype of *Cryptococcus gattii* caused the cryptococcosis outbreak on Vancouver Island (British Columbia, Canada). *Proc Natl Acad Sci U S A* **101**, 17258-17263, doi:0402981101 [pii]10.1073/pnas.0402981101 (2004).

7 Voelz, K. *et al.* Transmission of hypervirulence traits via sexual reproduction within and between lineages of the human fungal pathogen *Cryptococcus gattii*. *PLoS Genet* (2013).

8 Byrnes, E. J., 3rd *et al.* Emergence and pathogenicity of highly virulent *Cryptococcus gattii* genotypes in the northwest United States. *PLoS Pathog* **6**, e1000850, doi:10.1371/journal.ppat.1000850 (2010).

9 Hagen, F. *et al.* Autochthonous and dormant *Cryptococcus gattii* infections in Europe. *Emerg Infect Dis* **18**, 1618-1624, doi:10.3201/eid1810.120068 (2012).

10 Pereira, C. B. *et al.* Evaluation of laccases and melanization in clinical and environmental *Cryptococcus neoformans* samples by non-denaturing PAGE. *J Med Microbiol* **58**, 563-566, doi:58/5/563 [pii]10.1099/jmm.0.007716-0 (2009).

11 Meyer, W., Castaneda, A., Jackson, S., Huynh, M. & Castaneda, E. Molecular typing of IberoAmerican *Cryptococcus neoformans* isolates. *Emerg Infect Dis* **9**, 189-195 (2003).

12 Trilles, L. *et al.* Regional pattern of the molecular types of *Cryptococcus neoformans* and *Cryptococcus gattii* in Brazil. *Mem Inst Oswaldo Cruz* **103**, 455-462, doi:S0074-02762008000500008 [pii] (2008).

13 Chaturvedi, S., Ren, P., Narasipura, S. D. & Chaturvedi, V. Selection of optimal host strain for molecular pathogenesis studies on *Cryptococcus gattii*. *Mycopathologia* **160**, 207-215, doi:10.1007/s11046-005-0162-7 (2005).

14 Schmeding, K. A., Jong, S. C. & Hugh, R. Sexual compatibility between serotypes of *Filobasidiella neoformans* (*Cryptococcus neoformans*). *Curr. Microbiol.* **5**, 133-138 (1981).

15 Voelz, K., Johnston, S. A., Rutherford, J. C. & May, R. C. Automated analysis of cryptococcal macrophage parasitism using GFP-tagged cryptococci. *PLoS ONE* **5**, e15968, doi:10.1371/journal.pone.0015968 (2010).

16 Fraser, J. A. *et al.* Same-sex mating and the origin of the Vancouver Island *Cryptococcus gattii* outbreak. *Nature* **437**, 1360-1364, doi:nature04220 [pii]10.1038/nature04220 (2005).

17 Espinel-Ingroff, A. *et al.* Collaborative comparison of broth macrodilution and microdilution antifungal susceptibility tests. *J Clin Microbiol* **30**, 3138-3145 (1992).

18 Lazera, M. S. *et al.* Natural habitat of *Cryptococcus neoformans* var. *neoformans* in decaying wood forming hollows in living trees. *J Med Vet Mycol* **34**, 127-131 (1996).

19 Perfect, J. R., Lang, S. D. & Durack, D. T. Chronic cryptococcal meningitis: a new experimental model in rabbits. *Am J Pathol* **101**, 177-194 (1980).

20 Boekhout, T. *et al.* Hybrid genotypes in the pathogenic yeast *Cryptococcus neoformans*. *Microbiology* **147**, 891-907 (2001).

21 Kwon-Chung, K. J., Edman, J. C. & Wickes, B. L. Genetic association of mating types and virulence in *Cryptococcus neoformans*. *Infect Immun* **60**, 602-605 (1992).

22 Litvintseva, A. P. & Mitchell, T. G. Most environmental isolates of *Cryptococcus neoformans* var. *grubii* (serotype A) are not lethal for mice. *Infect Immun* **77**, 3188-3195, doi:IAI.00296-09 [pii]10.1128/IAI.00296-09 (2009).

23 Litvintseva, A. P. *et al.* Evidence that the human pathogenic fungus *Cryptococcus neoformans* var. *grubii* may have evolved in Africa. *PLoS ONE* **6**, e19688, doi:10.1371/journal.pone.0019688PONE-D-11-02521 [pii] (2011).
